# A review of enhanced recovery after surgery in kidney and liver transplantation and outline of the Newcastle ERAS protocols

**DOI:** 10.3389/frtra.2026.1704028

**Published:** 2026-05-28

**Authors:** E. Bonner, C. Scuffell, F. Dowen, L. MacDougall, J. Fabes, D. M. Manas, A. Amer

**Affiliations:** 1 Institute of Transplantation, Newcastle upon Tyne Hospitals NHS Foundation Trust, Newcastle upon Tyne, United Kingdom; 2 NHS Blood and Transplant, Bristol, United Kingdom; 3 University Hospitals Plymouth NHS Trust, Plymouth, United Kingdom; 4 Faculty of Health, University of Plymouth, Plymouth, United Kingdom

**Keywords:** enhanced recovery after surgery, ERAS, kidney transplantation, liver transplantation, protocol, review

## Abstract

Although Enhanced Recovery after Surgery (ERAS) is now common practice across various surgical specialties, the adoption of its principles has only recently gained momentum in liver and kidney transplantation. The publication of relevant ERAS guidelines in these specialties has undoubtedly facilitated the assimilation of evidence in support of ERAS components, the paucity of which had been viewed as a barrier to the implementation of ERAS in transplantation. In conjunction, institutionally-developed ERAS pathways have provided pragmatic examples of how these principles can be adopted within transplant practice. In this article, we summarise the current recommendations and underlying evidence for key components of an enhanced recovery programme in kidney and liver transplantation, with reference to contemporary national guidance, and outline the Newcastle ERAS protocols in these specialties as illustrative models of implementation.

## 1 Introduction

Originally pioneered by Henrik Kehlet for colorectal surgery in the 1990s, the concept of a multimodal rehabilitation programme structured around the perioperative period has evolved into Enhanced Recovery After Surgery (ERAS) programmes across a wide variety of surgical specialties (1, 2). Such programmes have consistently demonstrated reduced inpatient length of stay and improved patient satisfaction (3). Despite this, ERAS has not been widely adopted within the field of transplantation. Reasons for the slow uptake likely include embedded cultural practices within transplant units, concerns over the physiological complexity of transplant recipients, and the limited transplant-specific evidence in support of ERAS (4).

There is now a growing drive towards patient-centred, integrated care in many modern healthcare systems and an increasing interest in ERAS within the transplant community, particularly in kidney and liver transplantation. Various ERAS guidelines for kidney and liver transplant recipients have recently been published wherein current supportive evidence is appraised (5–7). These guidelines have begun to address some of the barriers impeding the wider implementation of ERAS in transplantation.

Alongside international recommendations, national pathways such as those produced by NHS Blood and Transplant (NHSBT) have further contextualised ERAS within UK transplant practice. Furthermore, published centre-specific protocols serve as pragmatic implementation models that align the guidance with real-world clinical delivery. The Newcastle ERAS protocols for kidney and, more recently, liver transplant recipients have transformed local transplant practices and served as one of the working models for the NHSBT national ERAS programme.

In this article, we aim to summarise the current published recommendations and underlying evidence in support of key components of an enhanced recovery programme in kidney and liver transplantation. We also outline the respective Newcastle ERAS protocols in these specialties for reference.

## 2 ERAS in renal transplantation

### 2.1 Global evolution of ERAS practice in kidney transplantation

**Figure 1:**
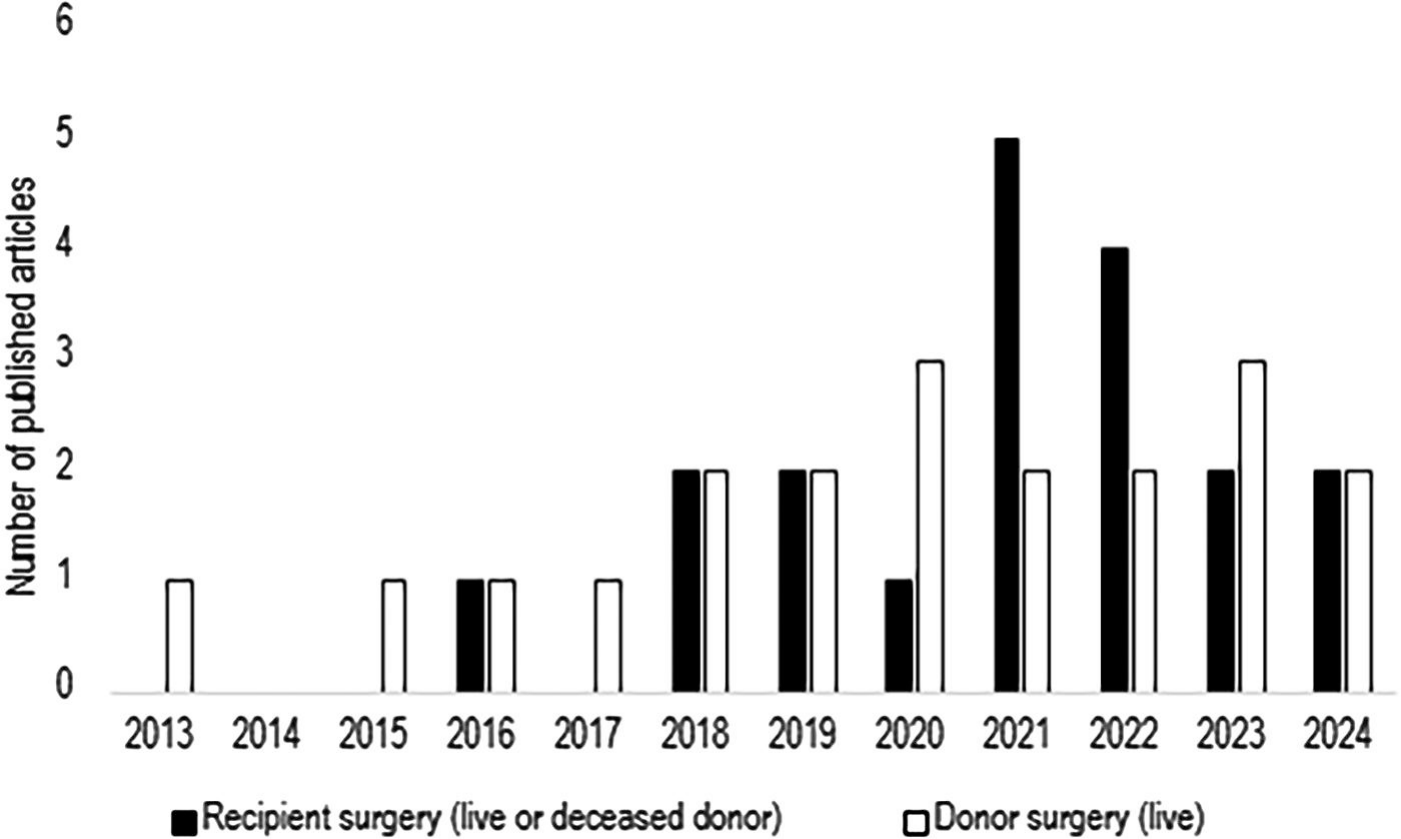
Trends in published literature on ERAS in kidney transplantation for both recipient and donor surgery.

Interest in enhanced recovery after kidney transplantation has seen a gradual rise over the past decade. Published reports on ERAS in kidney transplantation have increased steadily over the past decade, with initial work focusing on living donor surgery before expanding to transplant recipients (Figure 1). Despite this growth, renal transplantation still represents a relatively small proportion of the ERAS literature compared to other surgical specialties (8). Studies evaluating local enhanced recovery pathways in kidney transplantation have been published by centres in the UK (9, 10), USA (11–13), Italy (14), Poland (15) and Australia (16). A UK national survey demonstrated that six transplant centres had implemented a formal ERAS pathway for kidney transplant recipients, although deceased donor recipients were excluded from half of these pathways at the time of analysis (4). More recently, NHSBT has produced national guidance for adult kidney transplant recipients, providing a structured framework to support wider implementation across UK transplant centres (5).

### 2.2 The Newcastle ERAS protocol for kidney transplant recipients

The Newcastle kidney transplant ERAS protocol was introduced in 2021 for living donor recipients and subsequently extended to all recipients following a pilot phase which demonstrated feasibility, safety and excellent feedback from patients and clinical staff, in addition to a reduction in opioid use, postoperative nausea and vomiting scores and overall length of hospital stay (17). The protocol evolved from earlier work by the Belfast and Sheffield kidney transplant groups (9, 10) and took insight from a survey of contemporary practice amongst kidney transplant units in the UK (4).

The protocol incorporates a number of key components, which are addressed in individual sections of this review and further outlined in Table 1 and the Supplementary Material.

**Table 1:** Outline of the Newcastle ERAS protocol for kidney transplant recipients.

Component	Description
Preoperative patient education and counselling	Provide in clinic for living donor recipients Revisit or deliver on admission for all potential kidney transplant recipients Interactive patient journal to be provided at the time of patient counselling*
Prehabilitation on waiting list**	Identify and address patient challenges prior to surgery (frailty, social issues… etc) Provide advice and signpost to resources
Fluid management	Preoperatively
	Formal fluid balance assessment within 24 h of admission to estimate target weight Intraoperatively
	Aim for euvolaemia. Usually < 2 L fluid intraoperatively using balanced crystalloid fluids e.g., Hartmann's solution Assess fluid responsiveness using PPV or SVV from LiDCO via the arterial line. Aim < 8% Aim for MAP > 70 mmHg Use metaraminol as required for blood pressure support Standard lines: Arterial line: routine for monitoring and blood sampling. Check ABG post intubation for baseline acid/base status. Central venous line: not routinely necessary for monitoring. Place if anticipated need for vasopressor intra/postoperatively Post-operatively in recovery
	Fluid replacement: IVT input = output If urine output 40 mls/h or less give IVT @ 40 mL/h Target MAP > 70 mmHg If MAP low, assess fluid status and consider a fluid challenge of up to 12 mL/kg bolus crystalloid in 250 mL boluses Use peripheral vasopressors if poor response to fluid boluses and consider prolonging PACU admission If recipient is staying in post anaesthetic care unit (PACU) overnight, follow post-operative ward guidance (see below) Encourage to start drinking fluids as soon as able post anaesthetic Postoperatively after transfer from PACU to ward or level 2/3 care
	Daily weight before breakfast IV fluid replacement IVT Input = Output in the immediate postoperative period If urine output 40 mls or less, keep IVT at 40 mL/h Aim to cap IVT at a 3 L limit in 24 h to avoid driving polyuria, unless systolic <110 mmHg Stopping IVT For living donor recipients: Aim to stop IVT at breakfast time on post-op day 1 For deceased donor recipients: Stop IVT when able to drink (aim to stop approximately 12 h postoperatively) If vomiting/ileus then continue IVT based on fluid balance assessment General principles Detailed fluid balance assessment to be undertaken at least twice daily to avoid dehydration or overload. Avoid postoperative weight gain of over 3–5 L (5% of preoperative dry weight) Give patients a daily oral fluid target at morning ward round based on fluid balance assessment. Aim to cap at 3 L
Analgesia	TAP catheter insertion intraoperatively. 10 mL 0.5% levobupivacaine is given via TAP catheter as a bolus followed by 5 mL/h of 0.25% levobupivacaine via a fixed rate pump PCA for breakthrough pain (removed within 12–24 h postoperatively) Avoid long-acting opioids PCA replaced with PRN Oxynorm (plus regular oxynorm—only if struggling with pain) Regular pain team review as required
Abdominal drains	If drain required, aim for 1 non-suction drain Consider drain removal on day 2 if serous or haemoserous, and output is less than 100 mL/24 h (surgical decision) If output is over 100 mL/24 h on day 2, measure drain fluid creatinine and remove drain if result is reassuring.
Urinary catheter removal*	On admission perform post-micturition bladder scan on ward If residual volume <150 mL remove catheter on day 2 unless: visible haematuria urine output >5 L/24 h instructed to leave *in situ* by operating surgeon Delay catheter removal until day 5 in the case of: pre-op urine residual volume > 150 mL No residual volume is recorded and patient has either of the following: diabetes underlying urological pathologies
Oral diet	Oral fluids and diet a few hours after recovery from anaesthesia as tolerated
Early mobilisation*	Mobility programme will be based on the group stratification based on mobility score and physio assessment on day 1 Within the first 24 h—aim to sit out in chair at least once Day 1—Formal physiotherapy assessment Breathing exercises Out in chair for all meals Target number of hours in chair (depending on group stratification) Two-four walks (depending on group stratification) Day 2 onwards—(depending on group stratification) Target number of walks Sit in chair for each meal Target number of hours in chair Individualised exercise programme Day 5—Repeat mobility assessment by physios Individualised rehabilitation programme for discharge where appropriate.
Education and preparation for discharge	Admitting nurse to confirm any potential challenges to discharge and name of support person for transplant education. Education programme Self-medication training commenced from day 2 Transplant education provided on days 2 and 3 Discharge preparation Day 3—Identify date and time for TNL removal and confirm transport arrangements for discharge Day 4—Discharge medications to be prescribed and agreed on ward round. Discharge follow up plan and make arrangements with local centre

IVT, intravenous therapy; MAP, mean arterial pressure; PACU, post anaesthetic care unit; PCA, patient-controlled analgesia; PPV, pulse pressure variation; SVV, stroke volume variation; TAP, transversus abdominis plane; TNL, tunnelled neck line.

*See Supplementary Data for supporting material.

** A formal prehabilitation programme remains an aspiration impeded mainly by limited resources.

### 2.3 Key components of an ERAS programme in kidney transplantation

#### 2.3.1 Patient counselling and education

A key focus of ERAS programmes is patient empowerment through structured preoperative education and counselling, encouraging active participation in recovery. While data from major abdominal surgery demonstrates that preoperative education reduces psychological stress and anxiety, transplant-specific evidence remains limited (18, 19). Tan et al. recommended structured preoperative education and counselling for renal transplant recipients, covering postoperative care regimens, potential complications, and strategies for self-management (20). NHSBT guidance further defines ERAS education and counselling for adult kidney transplant recipients to include an introduction to ERAS principles, expectation management, identification of recovery barriers, and provision of standardised educational materials (5). Education should begin at the earliest feasible opportunity and be reinforced at clinic appointments, waiting list reviews and on admission for transplantation, reflecting the current focus on early and frequent patient education intervention prior to major surgery (21).

Within the Newcastle kidney transplant ERAS protocol, counselling begins during the initial transplant assessment visit and is repeated on admission for transplantation (Table 1). Patients are also provided with a journal that outlines key recovery milestones and encourages active participation in postoperative rehabilitation (see Supplementary Material).

#### 2.3.2 Prehabilitation and postoperative rehabilitation

Prehabilitation before surgery should be a multimodal approach aiming to improve patients' physical, psychological and social wellbeing with an increase in resilience in all sectors. A combination of physical exercise, nutritional support and cognitive and psychosocial interventions are used to ensure patients are optimised for both their perioperative journey and rehabilitation after surgery (22). End stage renal failure and the associated physiological effects lead to a significant decline in physiological systems with associated frailty and sarcopenia (23, 24). For many patients with end stage kidney disease, particularly those on dialysis, a sedentary lifestyle becomes habit and the associated anxiety and depression whilst on transplant lists can be significant barriers to improving physical and psychological wellness (25). Prehabilitation programmes in this cohort aim to address these issues and optimise patients not only to achieve good clinical outcomes, but also to introduce lifestyle changes that may be carried on after transplantation for improved long term health outcomes.

The Kidney Disease: Improving Global Outcomes (KDIGO) recommendations for kidney transplant candidates include referral to smoking cessation programmes for patients using tobacco products and consideration of dietary counselling or referral for bariatric surgery for those with BMI >35 kg/m^2^ (26). The European Society of Organ Transplantation (ESOT) consensus statement published in 2023 strongly supports prehabilitation for solid organ transplant candidates, and data from the recently published FRAILMar RCT demonstrates that structured exercise and nutritional intervention results in significant improvements in physical performance measures and frailty indices for patients awaiting kidney transplantation (27). These findings strengthen the case for structured prehabilitation as a feasible and clinically meaningful component of kidney transplant ERAS pathways.

The feasibility of prehabilitation in kidney transplant recipients is well described and can be implemented for most patients, even within the confines of dialysis (28). These may be hospital- or home-based. Hospital-based physiotherapy programmes have demonstrated decreased length of stay and importantly no adverse effects (28). Similarly, home-based exercise models, often supported by digital platforms, can lead to improved outcomes and show an increased acceptance and higher adherence rates than physiotherapy or hospital-based programmes (29–31). However, inequity of access remains a challenge in the UK due to funding constraints (4, 32).

Early mobilisation following surgery is an integral part of an ERAS programme and is associated with reduction in complications, hospital length of stay and overall recovery times (20, 22). Postoperative mobilisation should be viewed as a continuation of prehabilitation, reinforcing the concept of a continuous “prehab-to-rehab” trajectory.

The Newcastle kidney transplant ERAS protocol integrates prehabilitation and postoperative rehabilitation as a process that extends from the waiting list period through the postoperative ward stay. Candidates are assessed for frailty, nutritional deficits and physical deconditioning during transplant evaluation, with referral to physiotherapy or dietetic services where appropriate. Following transplantation, the pathway emphasises mobilisation beginning on the first postoperative day, with early physiotherapy review where required. Patients are encouraged to sit out of bed within the first 24 h and progress to assisted ambulation thereafter, with mobility targets reviewed during daily ward rounds (Table 1).

#### 2.3.3 Fluid monitoring and replacement

There is evidence to suggest that optimisation of intraoperative fluid status may reduce the risk of graft dysfunction with associated improvements in long-term kidney survival, length of stay and healthcare costs (33–36). Conversely, excessive fluid administration promotes peripheral, visceral and pulmonary oedema, impaired microcirculation and potential graft dysfunction (37–40).

Kidney allografts lack autonomic autoregulation following denervation, rendering perfusion dependent on systemic pressure and flow (41). While there is some evidence that higher mean arterial pressure (MAP) may yield improved graft outcomes, there is nothing robust on which to propose MAP targets (42, 43). Furthermore, there is no evidence base to guide the choice of inotropic or vasopressor agents to achieve this. It is likely that any approach that maintains a sensible MAP, mindful of volume status and both donor and recipient baseline MAP, will yield good graft perfusion.

Goal-directed haemodynamic therapy (GDHT), supported by cardiac output monitoring, is recommended by NICE for major surgery and has been shown to reduce complications and length of stay in bowel, liver and pancreatic surgery (44). In kidney transplantation, GDHT results in reduced cardiovascular complications, postoperative ileus, reductions in pulmonary oedema, improved mobilisation and no increase in delayed graft function (10, 16, 45–50).

Cardiac output monitoring is better than central venous pressure (CVP), at determining fluid responsiveness in renal transplantation (51). Balanced crystalloid solutions result in a better plasma acid-base profile and non-inferior potassium concentration when compared to 0.9% sodium chloride solution (52, 53). However, it remains unclear whether this translates into clinical benefit, particularly in relation to graft outcomes. The recent recommendations by the American Society of Anesthesiologists (ASA) on fluid management during kidney transplantation support the preferential use of balanced crystalloid solutions. There is no evidence to support any advantage for using albumin over crystalloid solutions, and the use of hydroxyethyl starch (HES) solutions is cautioned against due to the increased risk of renal injury (54).

Interestingly, a recent survey of anaesthetic practice in the UK showed significant heterogeneity in fluid replacement and blood pressure management for deceased donor kidney transplant recipients (55). Centres utilising ERAS programmes in renal transplantation aim to stop intravenous fluid therapy once the patient is tolerating oral fluids, as early as 6 h postoperatively, and direct patients with fluid goals (9).

Within the Newcastle kidney transplant ERAS protocol, intraoperative haemodynamic management prioritises maintenance of adequate renal graft perfusion while avoiding excessive fluid administration. Balanced crystalloid solutions are used as the primary replacement fluid and haemodynamic targets are guided by dynamic monitoring indices such as pulse pressure variation, with a target MAP >70 mmHg. Postoperatively, intravenous fluids are titrated against urine output and discontinued early once oral intake is established, usually within the first 12 h of surgery (Table 1).

#### 2.3.4 Perioperative analgesia

Key aims for analgesia in ERAS for kidney transplantation are effective pain control, early mobilisation and opioid minimisation. High perioperative opioid exposure after kidney transplantation is associated with graft failure and an increased mortality (56). Regional analgesia such as Transvs. Abdominis plane (TAP) blocks and wound catheters in combination with robust multimodal oral analgesia can reduce the need for patient-controlled analgesia (PCA) (20, 22, 57). NSAIDs are avoided due to nephrotoxicity concerns while gabapentinoids should be used cautiously in dialysis patients due to concerns over increased hospitalisation and mortality when used in combination with opioids (58). Intrathecal morphine is effective in reducing postoperative opiate requirement but can induce itching post op and care needs to be taken due to the potential for respiratory depression (22). Similar to fluid replacement and blood pressure management protocols, considerable variation exists amongst UK kidney transplant centres over pain management for recipients, particularly with regards to PCA composition and the use of regional analgesic adjuncts (55).

The Newcastle kidney transplant ERAS protocol uses a structured opioid-sparing analgesic strategy designed to facilitate early mobilisation and recovery. Regional analgesia forms the cornerstone of the pathway, with TAP catheter placement and continuous local anaesthetic infusion used routinely following transplantation. Short-duration opioid PCA is used for breakthrough pain and is typically discontinued within the first 12–24 h once oral intake is tolerated and oral analgesia is established (Table 1).

#### 2.3.5 Selective indwelling line insertion and early removal

There is paucity of evidence to support the routine insertion of central venous catheters (CVC) at the time of kidney transplantation (54). Central venous pressure monitoring has been shown to be unreliable for assessing fluid responsiveness, and target pressures vary considerably between transplant centres that monitor CVP intraoperatively (55, 59, 60). CVC insertion can still be considered in cases where access is difficult or when centrally administered intravenous medication is required (10). NHSBT guidance supports selective rather than routine CVC use (5).

The insertion of a urinary catheter is a widely accepted practice in kidney transplantation. However, there is considerable variation in practice with regards to the timing of urinary catheter removal (4). While high quality randomised controlled studies investigating the clinical impact of early urinary catheter removal in kidney transplant recipients are currently lacking (61), the published experience from transplant centres that practice early removal of urinary catheters suggests that it is safe, even as early as day 1 following kidney transplantation (13, 62–64). Indeed, catheter removal within 48 h of transplantation is recommended in uncomplicated cases based on recent NHSBT guidance (5). A randomised controlled trial is currently underway investigating the safety of early urinary catheter removal after renal transplantation (ELUCATR study—ClinicalTrials.gov ID NCT04815954). The trial is expected to be completed in 2026 and may provide crucial evidence in this regard.

Similarly, there are no high quality evidence on the efficacy of drain insertion in kidney transplantation. A recent meta-analysis of four published retrospective cohort studies concluded that drain placement offers no benefit in terms of wound complications and reintervention rates (65). Notwithstanding the inherent limitations of retrospective data analysis and the potential for selection bias in the included studies, the evidence argues the case for selective drain insertion in kidney transplantation, which is reflected in the NHSBT guidance (5). Centres that have reported routine drain insertion in ERAS protocols describe drain removal between day 2 and 4 if output is below 100 mL/day (10, 15, 16).

The Newcastle kidney transplant ERAS protocol adopts a selective approach to invasive line and drain insertion following kidney transplantation. Central venous catheters are used only where clinically indicated rather than routinely. Urinary catheters are typically removed within 48 h in uncomplicated recipients once adequate urine output and patient mobilisation are established. Surgical drains are inserted selectively and are also removed within 48 h if output is low and of serous content. Further details on criteria for early catheter and drain removal can be found in Table 1 and Supplementary Material 1.4.

#### 2.3.6 Carbohydrate loading and perioperative nutrition

There is limited evidence for the use of preoperative carbohydrate-rich clear liquids and reduction of preoperative fasting in renal transplantation. Preoperative carbohydrate-containing clear liquids, given up to 2 h prior to surgery, have been included in the ERAS Society's recommendations to reduce postoperative insulin resistance, improve perioperative glucose control and minimise preoperative fasting (66, 67). One UK centre has reported the safe use of preoperative carbohydrate drinks in non-diabetic kidney transplant recipients as part of a multimodal ERAS programme (10). The recommended dose in general surgical procedures is 100 g of carbohydrate the night before surgery and 50 g 2 h before surgery in non-diabetic patients (68). This is usually delivered in 200 mL volumes per 25 g of carbohydrate and therefore caution should be taken in potential kidney transplant recipients where preoperative fluid restrictions apply.

Kidney transplant recipients are at high risk of protein energy malnutrition in the perioperative period. Postoperative oral nutrition should therefore be commenced at an early stage following transplantation, which is achievable in most cases as gut function is rapidly restored. This is supported by guidance from the European Society for Clinical Nutrition and Metabolism (ESPEN) (69, 70).

Nutritional management within the Newcastle kidney transplant ERAS protocol focuses on minimising perioperative fasting and promoting early postoperative oral intake. Although the use of carbohydrate loading is not standard practice at our centre, prolonged fasting prior to transplantation is avoided and a “sip till send” approach is routinely implemented before surgery. Oral intake is resumed as soon as safely possible following surgery, typically within the first 12 h of surgery. Nasogastric tubes are not routinely used and are reserved only for specific clinical indications (Table 1).

## 3 ERAS in liver transplantation

### 3.1 Global evolution of ERAS practice in liver transplantation

**Figure 2:**
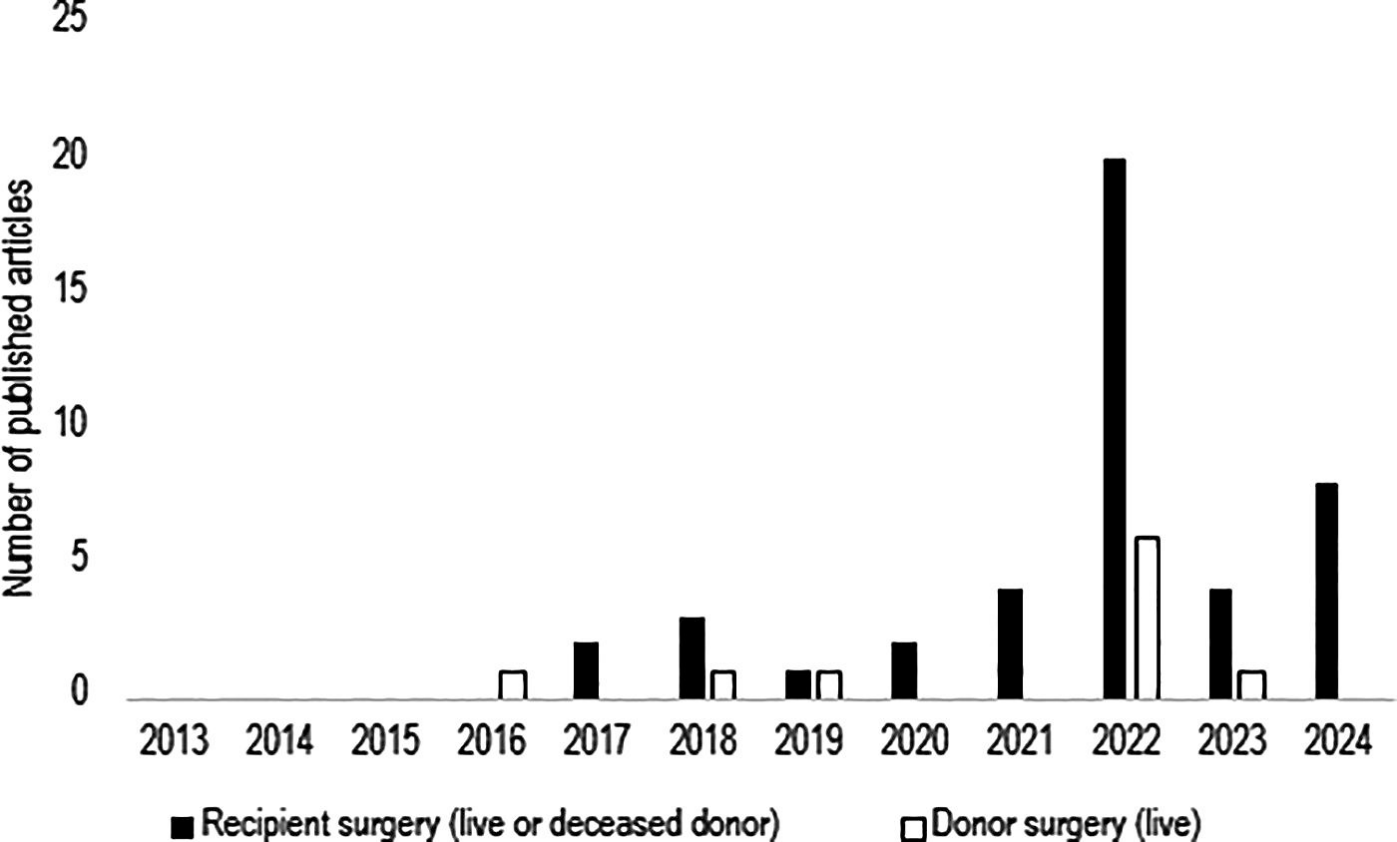
Trends in published literature on ERAS in liver transplantation for both recipient and donor surgery.

While reports of early extubation after liver transplantation date back as early as 1990 (71), structured enhanced recovery pathways for liver transplantation have only begun to emerge over the past decade, around the same time as ERAS publications in kidney transplantation. However, in contrast to kidney transplantation, recipient ERAS pathways have been more prominent than donor pathways in liver transplantation. Figure 2 demonstrates the increasing volume of publications related to ERAS in liver transplantation, with a notable peak in 2022 reporting the recommendations of the ERAS4OLT international consensus conference (7). Additional guidance from the ERAS Society was published in the same year, consolidating available evidence and expert opinion into practical perioperative recommendations for liver transplant programmes (6). To date, recipient ERAS pathways have been reported in liver transplant centres from various countries across the world including Turkey (72); China (73–75), India (76), Denmark (77), Spain (78), USA (79, 80) and France (81). In the UK, ERAS pathways have emerged in recent years and national guidance on ERAS in liver transplantation was released by NHSBT with the aim to standardise perioperative care and reduce unwarranted variation across transplant centres.

### 3.2 The Newcastle ERAS protocol for liver transplant recipients

The Newcastle ERAS protocol for liver transplantation was introduced in 2024 following the earlier implementation of an ERAS pathway for kidney transplantation. The protocol draws on international ERAS4OLT recommendations (7) while adapting them to local multidisciplinary practice. Although this protocol has only recently been introduced, it has provided a useful example of a functioning protocol which, along with other existing pathways, has informed the development of the recently released NHSBT guidance on ERAS in liver transplantation. Table 2 outlines the key components of the Newcastle ERAS protocol for liver transplant recipients, which are further detailed in the relevant sections of this review.

**Table 2:** Outline of the Newcastle ERAS protocol for liver transplant recipients.

Component	Description
Preoperative patient counselling and education	Introduced at time of consent, provided in clinic and revisited on admission for all potential liver transplant recipients Interactive patient journal to be provided at the time of patient counselling*
Prehabilitation on waiting list	Identify and address patient challenges prior to surgery (frailty, social issues… etc) Provide advice and signpost to resources Aim to introduce prehabilitation as resources allow to include: fitness optimisation, nutritional optimisation, social work and counselling input, onward referral to specialist services as indicated (e.g., smoking cessation, substance misuse)
Nutrition	Avoid prolonged fasting (>6 h). Offer snack prior to leaving home on admission. and follow “Sip till send” approach Routine use of wide bore nasogastric tube Avoid routine use of nasojejunal tube (only to be inserted in selected cases) Commence oral diet within 24 h post extubation Aim to remove nasogastric tube within 24 h of extubation (If beginning to tolerate oral diet) If not expected to eat within 48 h, commence enteral feeding
Surgical approach	Consider “reverse L” incision in appropriate cases
Abdominal drains	Consider selective insertion of surgical drains (one hilar drain may suffice) Aim to remove surgical drain by day 3 following uncomplicated liver transplant procedures if not bile stained, and output is less than 1,000 mL/24 h
Perioperative anaesthesia management	Set-up and monitoring to include Peripheral IV canula for induction +/− peripheral wide bore ETT Bypass lines if required Large bore, multi-lumen CVC Arterial line—consider proximal, consider two Nasogastric tube—wide bore, drainage tube Urinary catheter Oesophageal temperature monitoring Thermoregulation—upper and lower forced air warmers (consider additional warming e.g., foil hats if temperature maintenance is challenging) CO: non-invasive monitoring as standard. Consider invasive (PAC) on individual basis and with cardiac backup for both anaesthetic and ODP expertise if required Infusions: Remi/fentanyl—consider swapping for fentanyl towards end of case as can be transferred into PCA once extubated NMB—infusion or consider intermittent with TOF monitoring Calcium—tailor infusion according to ABG—consider reduced rate and stop at reperfusion Tranexamic Acid—individual consideration based on findings of fibrinolysis on TEG Noradrenaline Volatile with MAC monitoring Ventilation Aim PaO2 10–14 kPa Vt 6–8 mls/Kg IBW tailored PEEP Point Of Care Testing ABG/TEG at discretion of anaesthetist Blood glucose management Insulin dependent: Actrapid sliding scale (follow local ITU protocol Diet/tablet controlled: Commence sliding scale if blood glucose >10 mmol/L for 2 consecutive hours. Fluid management Restrictive intraoperative fluid management MAP > 60
Proactive extubation	Aim to extubate within a maximum of 4 h from arrival to ITU CPAP ASB on arrival or within 30 min Fentanyl 1–2 mL/h (100–200 mcg/h) Propofol to maintain light tube tolerance only Criteria for extubation to include BE less than −8 (and falling or plateau) lactate less than 5 (and falling or plateau) noradrenaline 4/50 < 5 mls/h drain output less than 300 mls in 2 h following insertion patient obeying commands
Pain management	Intraoperative opiate infusion (fentanyl/remifentanil) Magnesium 50 mg/Kg intraoperatively Commence fentanyl infusion prior to transfer to ITU (unless immediate extubation planned) If extubation imminent, bolus fentanyl and extubate onto fentanyl PCA Extubation Aim to remove PCA once tolerating oral or enteral diet and pain is under control. Replace with regular oramorph (or oxynorm if renal function impaired) Regular pain team review as required post -operatively
Mobility*	Mobility assessment within 24 h of extubation by qualified physiotherapist Aim to stand and march and/or sit out in chair at least once within the first 24 h of extubation In ITU—Daily physiotherapy assessment by qualified physiotherapist day 1–3 with handover to band 4 once deemed suitable Individualised bespoke programme ‘prescribed’ daily to include breathing exercises, time in chair for meals, number of hours in chair, number of walks. Daily progress to be charted in patient journal. Patients to be encouraged to record own progress, supported by ITU nursing/ physio staff as appropriate. On transfer to ward—Continued individualised exercise programme to include additional exercises as appropriate. Daily progress to be charted on mobility chart, patients to be encouraged to record own progress in journal, assisted by nursing/physio staff Repeat mobility assessment by physios including stair assessment ahead of discharge as appropriate. Individualised rehabilitation programme provided for discharge, with onward referrals for ongoing rehab as needed
Urinary catheter	Target catheter removal on day 2 (if meets criteria) dischargeable from level 2/3 care not oliguric/anuric able to mobilise or use urine bottles
Education and preparation for discharge	Education programme Day 1 following transfer to ward—self-medicating education provided. Day 2 following transfer to ward—delivery of post-transplant education. Day 3 following transfer to ward—consolidation of post-transplant education (repeat as needed). Discharge preparation Confirm transport arrangements for discharge 48 h prior to anticipated day of discharge Discharge medications to be prescribed and agreed on ward round with consultants the day before expected discharge. Medications ordered from pharmacy the day before expected discharge Document day, time and place of follow up in patient journal. Ensure social support is in place if required Ensure contact details for clinical support are provided to patient (liver transplant coordinator during working hours, ward out of hours)

ASB, assisted spontaneous breathing; ABG, arterial blood gas; BE, base excess; CO, cardiac output; CPAP, continuous positive airway pressure; CVC, central venous catheter; ETT, endotracheal tube; IBW, ideal body weight; MAC, mean alveolar concentration; MAP, mean arterial pressure; NMB, neuromuscular blockade; PAC, pulmonary artery catheter; PCA, patient controlled analgesia; ODP, operating department assistant; PEEP, positive end expiratory pressure; TEG, thromboelastography; TOF, train of four.

* See Supplementary Data for supporting material.

### 3.3 Key components of an ERAS programme in liver transplantation

#### 3.3.1 Patient counselling and education

Patient education promotes preparedness, empowerment and management of expectations, all of which are key elements of enhanced recovery after liver transplantation. A recent review concluded that living liver donors who received pre-operative counselling experienced improved outcomes, including lower levels of postoperative pain and fatigue, shorter recovery times, and less postoperative anxiety (82). In liver transplant recipients, inadequate patient understanding of the transplant process has been shown to be associated with reduced treatment adherence and poorer engagement with postoperative care (83).

Guidance published by the ERAS society recommends that patients on the waiting list for liver transplantation should receive dedicated, multidisciplinary educational counselling (6). Although a universally agreed framework for ERAS education in liver transplantation has not yet been defined, it is reasonable to assume that this should mirror that of kidney transplantation and other ERAS programmes, and address perioperative expectations, postoperative recovery milestones, potential complications and lifestyle considerations following transplantation (6).

In line with the kidney transplant protocol, the Newcastle liver transplant ERAS protocol introduces structured counselling delivered by the multidisciplinary transplant. Rather than relying on a single educational interaction, the pathway uses repeated contact points and an interactive patient journal to establish expectations around early mobilisation, early resumption of oral intake and proactive participation in rehabilitation following transplantation (See Table 2 and Supplementary Material). Involvement of carers and family members is encouraged as part of the counselling process, recognising their important role in supporting recovery and long-term adherence to treatment (84).

#### 3.3.2 Prehabilitation and postoperative rehabilitation

Patients presenting for liver transplantation frequently demonstrate frailty, sarcopaenia and malnutrition as a consequence of advanced liver disease. A meta-analysis including nearly 700,000 patients undergoing major surgery demonstrated that frailty was associated with increased risk of major postoperative complication, readmission and discharge to a care facility as well as a four-fold increase in perioperative mortality (85). In liver transplantation, cardiorespiratory fitness measured by cardiopulmonary exercise testing has been shown to predict postoperative morbidity and mortality (86).

Prehabilitation programmes in ERAS pathways aim to reverse or mitigate frailty and sarcopaenia, and to optimise patients for their perioperative journey through a combination of exercise training, nutritional optimisation and psychological support. Multiple feasibility studies have demonstrated that both institutionally-based and home-based exercise programmes are safe and feasible for patients awaiting liver transplantation (87, 88).

The ERAS4OLT expert panel recommends prehabilitation for liver transplant candidates on the basis of absence of harm, and likelihood of short-term benefits in functional outcomes, despite the paucity of high quality evidence (89). The ERAS Society similarly acknowledges the lack of evidence for adapted physical therapy in liver transplantation in its recommendations (6).

Prehabilitation should not be limited to exercise and nutrition and should be addressed holistically. Anxiety and depression are prevalent in patients awaiting transplantation and can have a negative impact on outcomes (90). Whilst this can be positively affected by increased physical activity and improved nutrition, psychosocial aspects should be independently addressed in transplant candidates.

Early mobilisation and inpatient rehabilitation following liver transplantation are recommended by the ERAS4OLT expert panel who deemed it deemed safe, tolerable and feasible, with a potential positive impact on cardiorespiratory fitness (91). Likewise, the ERAS Society guidelines recommend early goal-directed interventions from the morning after liver transplantation and until discharge (6). Indeed, several centres have reported early ambulation as part of their ERAS pathways ranging from same day of surgery to postoperative day 2 (74–79). Rehabilitation measures should continue after discharge to build up functional capacity.

Similar to the kidney transplant protocol, prehabilitation in the Newcastle ERAS protocol begins with early identification of barriers to recovery while patients remain on the waiting list, including frailty, nutritional issues and social challenges (Table 2). Where resources allow, the pathway aims to extend beyond general advice to include fitness optimisation, dietetic input, counselling support and onward referral to specialist services such as smoking cessation or substance misuse teams.

Postoperative rehabilitation is described in the Newcastle liver transplant ERAS protocol in greater operational detail in Table 2. The mobility programme is stratified according to early physiotherapy assessment, with an initial aim to sit out within the first 24 h, followed by formal day 1 physiotherapy review, breathing exercises, chair-based recovery for meals, and a graded walking target according to functional status. From day 2 onwards, the pathway progresses to an individualised exercise programme with repeat physiotherapy assessment, including discharge planning and onward rehabilitation where required. This structure reinforces the concept of a continuous recovery trajectory extending from prehabilitation through postoperative rehabilitation.

#### 3.3.3 Proactive endotracheal tube extubation

Expedited extubation following major surgery has long been understood to improve outcomes (92). Historically, liver transplant recipients remained intubated for several hours following transplantation in order to allow stabilisation of physiological parameters and to ensure adequate graft function. However, the potential drawbacks of prolonged ventilation on both physiological and psychological parameters have led to an increased drive towards early extubation (within 4 h) (93).

Advances in surgical, anaesthetic and perioperative care should allow for earlier extubation of liver transplant recipients which is now considered a key component of ERAS pathways in liver transplantation. Early extubation has been associated with a reduction in pulmonary complications including ventilator-acquired pneumonia (94), decreased incidence of acute kidney injury (95, 96), improved graft function (97, 98) and shorter ITU and hospital lengths of stay (95–100).

Predictors of delayed extubation include high intraoperative transfusion requirements and high preoperative MELD or Childs Pugh C scores (96, 98, 101). The ERAS4OLT group recommend early extubation (within 4 h of surgery) in the majority of patients following a set of pre-determined criteria. If these criteria are met, then the risk of harm with expedited extubation is low (93).

Within the Newcastle liver transplant ERAS protocol, early extubation is considered the default approach where physiological parameters permit, with a target of extubation within 4 h of arrival to ITU. The protocol provides defined clinical criteria to guide extubation which are outlined in Table 2. In summary, the protocol specifies an early assisted spontaneous breathing strategy, light sedation only, and a set of physiological thresholds to support early extubation, including trajectory of base excess and lactate, low vasopressor requirement, reasonable drain output and ability to obey commands.

#### 3.3.4 Perioperative anaesthetic management of liver transplant recipients

Several comprehensive meta-analyses on the optimum anaesthetic practice in liver transplantation summarise the evidence and underlie the ERAS4OLT consensus guidelines in the respective domains (102–105).

A baseline standard of access includes arterial line insertion with central venous catheterisation with high flow venous access being established under direct ultrasonic vision (106). Discrepancy between peripheral and central arterial pressure should be considered, particularly in cases with anticipated cardiovascular instability and placement of proximal arterial catheters may be used to rationalise vasopressor use (102, 107).

Transoesophageal echocardiography is increasingly utilised in liver transplantation to assess both cardiac function and fluid responsiveness, and can be extremely useful in urgent situations of cardiovascular collapse. The level of risk associated with bleeding from oesophageal varices is accepted as low (108). Use of pulmonary catheter flotation devices is still widespread and considered safe (106), and local available expertise in either technique is necessary (102).

Intraoperative fluid management should be restrictive or on a replacement only basis, particularly during the hepatectomy phase (109–111). The incidence of AKI in transplant recipients is high and an independent predictor of mortality. Measures should be taken to avoid this, including maintaining a MAP above 60–65 mmHg, supported where necessary by low to moderate dose vasopressor therapy (112–114). Avoidance of hypervolaemia is important for optimisation of postoperative graft function and reduction in respiratory complications and associated critical care length of stay (104).

Point of care viscoelastic testing has become an important component of intraoperative coagulation management and has been associated with a reduction in transfusion requirements and improved haemodynamic stability (115, 116). Cell salvage is a useful tool to minimise non-autologous blood product transfusion and should be used as standard (105).

The Newcastle liver transplant ERAS protocol incorporates these principles through structured anaesthetic management. The protocol specifies large-bore central venous access, arterial monitoring with consideration of proximal or dual arterial lines, routine oesophageal temperature monitoring, active upper and lower body warming, and non-invasive cardiac output monitoring as standard, with pulmonary artery catheterisation reserved for selected cases. It also emphasises restrictive fluid management with a clear target MAP, careful calcium replacement and vasopressor support, and the use of viscoelastic testing to guide transfusion decisions and tranexamic acid administration. Details of these perioperative anaesthetic elements can be found in Table 2.

#### 3.3.5 Perioperative analgesia

Effective analgesia crucially interlinks with other postoperative components of an ERAS pathway such as early mobilisation, early resumption of oral diet and early removal of surgical drains and catheters. Advances in surgical technique with a trend towards less invasive incisions and reduction in intra-abdominal drain usage has led to the evolution of traditional, opiate heavy, post-operative analgesia regimens. Multimodal, balanced analgesia is recommended for the management of pain post liver transplantation (6, 7, 117). Opiate sparing techniques should be employed with the consideration of adjuncts such as anticonvulsants (gabapentin/pregabalin), ketamine, magnesium, paracetamol and local anaesthesia (117).

Regional blockade such as TAP blocks or wound catheter infusions may provide additional analgesic benefit, depending on the surgical incision. Some centres administer single dose intrathecal opiate to complement intravenous agents and regional blockade. Thoracic epidural analgesia is generally avoided due to the predictable coagulopathy associated with advanced liver disease and the risk of epidural haematoma (6).

With reference to the Newcastle liver transplant ERAS protocol, analgesia is planned as a continuum from theatre to extubation and early ward recovery. The pathway incorporates intraoperative opioid infusion with magnesium, followed by fentanyl infusion prior to transfer to ITU unless immediate extubation is anticipated. Where extubation is imminent, patients are transitioned directly onto fentanyl PCA, with a subsequent step-down to oral morphine or oxynorm once oral or enteral intake is established and pain is controlled. This staged multimodal analgesia approach is designed to support early extubation and mobilisation, and involves regular pain team reviews to avoid unnecessarily prolonged opioid exposure (Table 2).

#### 3.3.6 Selective indwelling line insertion and early removal

The placement of abdominal drains following liver transplantation remains widely practiced, mainly to detect and prevent the accumulation of intrabdominal fluids. However, several studies have challenged that principle, questioning the value of drains as a preventative tool (118–121). While some studies have shown that the need for postoperative paracentesis is higher in patients without surgical drains, particularly those with a pretransplant history of refractory ascites, the justification for routine drain insertion is debatable (122). When surgical drains are used, early removal is recommended where clinically appropriate. The ERAS4OLT expert panel suggests removal within five days following transplantation based on patient progress and drain output (123).

Central venous and urinary catheters are typically required during liver transplantation for haemodynamic monitoring and urine output measurement. However, prolonged use of indwelling catheters increases the risk of infective complications. Although the evidence for optimal timing of central venous and urinary catheter removal after liver transplantation is lacking, early catheter removal is one of the recommendations made by the ERAS4OLT expert panel (123).

The Newcastle liver transplant ERAS protocol takes a selective rather than routine approach to postoperative drains, recognising that one hilar drain may be sufficient in uncomplicated cases and aiming for removal by day 3 when output is not bile stained and remains below 1,000 mL per 24 h. Urinary catheter removal is targeted on day 2 provided the patient is out of level 2/3 care, not oliguric or anuric, and sufficiently mobile or able to use urine bottles. Daily clinical reviews are undertaken to rationalise indwelling lines, drains and catheter use if they are kept in beyond the target removal days (Table 2).

#### 3.3.7 Perioperative nutrition

Given the high risk of sarcopenia in liver transplant recipients, nutritional optimisation forms an essential component of both prehabilitation and postoperative recovery within an ERAS pathway (124). Early postoperative feeding has been shown to be safe in liver transplantation and is recommended by both the ERAS Society and ERAS4OLT guidelines. Ideally, this should be commenced within the first 12–24 h of transplantation if tolerated and if there are no surgical concerns. It is recommended that nasogastric tubes are removed on the first day after liver transplantation in non-ventilated patients (7) or indeed at the time of reversal of anaesthesia (6). The involvement of a dietician remains important to provide personalised perioperative nutritional input.

The Newcastle liver transplant ERAS protocol addresses nutrition across the full perioperative period. Preoperatively, it emphasises avoidance of prolonged fasting, recommends a snack before leaving home on admission, and follows a “sip till send” approach before surgery. Postoperatively, the pathway supports commencement of oral diet within 24 h of extubation, early removal of the nasogastric tube if oral intake is beginning to establish, and commencement of enteral feeding if oral intake is not expected within 48 h. It also avoids routine nasojejunal tube placement except in selected cases. Dietetic input is regularly sought to provide individualised nutritional support throughout the perioperative period (Table 2).

#### 3.3.8 Perioperative antimicrobial support

There is sufficient evidence to demonstrate the beneficial effect of antimicrobial prophylaxis in liver transplantation to reduce the morbidity and mortality associated with bacterial and fungal sepsis (125). While the choice of prophylactic antimicrobial therapy should be individualised according to patient requirements and centre policy, the duration of antibacterial prophylaxis should be limited to the first 24 h, based on existing evidence that shows no benefit from extended prophylaxis (126). Antifungal prophylactic therapy should be targeted at high-risk recipients, given that invasive fungal infections post liver transplantation are less common in the absence of risk factors but are of profound clinical consequences (125). Selective gut decontamination is not recommended and may in fact result in a higher rate of infective complications after liver transplantation (127).

The Newcastle liver transplant ERAS protocol supports antimicrobial stewardship by encouraging early progression of recovery milestones and timely removal of invasive devices that may contribute to infective risk. While antibacterial prophylaxis is routinely prescribed in the immediate perioperative period, prophylactic antifungal therapy is not routinely used and selective gut decontamination does not form part of the protocol.

## 4 Future direction

Although considerable progress has been made in the evolution of ERAS pathways in solid organ transplantation over the past decade, there is still much to be achieved. Specialities such as solid organ pancreas, heart and lung transplantation remain unchartered territory for enhanced recovery. ERAS for paediatric organ transplantation is also largely aspirational. These areas are currently being addressed through various workstreams of the NHSBT national ERAS programme which may pave the way for future implementation of ERAS in these fields.

Future research should focus on the impact of individual enhanced recovery components on relevant outcomes in relation to transplantation where high-quality data is lacking. Standardisation of ERAS protocols in transplantation will facilitate pathway audits and the collection of meaningful outcome data, and minimisation of variation in practice will benefit both clinician and patient.

Achieving this requires strong collaboration between transplant centres and professional bodies that champion the development of enhanced recovery pathways, and it is imperative that we cultivate multidisciplinary forums where hot topics and future advances in enhanced recovery in organ transplantation are addressed.
