# Supplementary material for: A review of enhanced recovery after surgery in kidney and liver transplantation and outline of the Newcastle ERAS protocols
Source: Front Transplant. 2026 May 28;5:1704028. doi: 10.3389/frtra.2026.1704028 (PMC13253640; doi:10.3389/frtra.2026.1704028)
Supplement: Supplementary file 1 [file Datasheet1.pdf]

The Newcastle upon Tyne Hospitals
NHS Foundation Trust

Enhanced recovery after surgery for liver transplant recipients

Patient journal

Healthcare at its best
with people at our heart

Introduction to enhanced recovery

Enhanced recovery after surgery (ERAS) is a programme designed to help you to recover more quickly from your transplant. We hope that this journal will help you to understand what to expect and feel more involved in what happens during your transplant in-patient journey.

To optimise your recovery after your transplant, it is important that you take an active role in your own care. Reaching certain targets can reduce the likelihood of complications. (For example, becoming mobile, sooner after surgery, can help to reduce the risk of chest infections, blood clots, and muscle wastage. Being better informed and taking an active role can help to improve your overall experience as you recover.

The journal is yours to keep and use to record your progress. Relatives, friends and the transplant team can help you to complete it if you find this difficult. By completing this journal each day, it will help the team to support you and ensure that your recovery is as smooth as possible. You can find some useful explanations of terms on page 27.

Don't worry if you do not meet all of your targets. Every patient is different and the team will support you to achieve your targets at a pace that's right for you. The team will adapt the programme where needed, to ensure that you receive high-quality care and we will not discharge you from hospital until both you and the transplant team feel that you are ready.

During your stay in critical care (ICCU)

The nurses will keep you well hydrated. You will usually be encouraged to start drinking as soon as you wake up. Ask the nurses if you are unsure about how much to drink.

When you wake up, the physiotherapists will assess you (usually within the first 24hours) and provide you with targets to aim for. They will support you with this and help you to record your progress on the opposite page. (See pages 21-26).

Eating enough food is essential to provide the fuel your body needs to heal. Sometimes we need to use the tube in your nose to provide nourishment if you are unable to eat. When you do start to eat, try to keep track and record what you are managing on the opposite page.

You will usually have a PCA (pain button) to keep you comfortable. It is important to be able to take deep breaths and cough. If you are struggling with this because of pain, or you are feeling sick, tell the nurses. We have many ways we can help.

We will try to remove your urinary catheter as soon as possible. Feel free to ask the team about this and if it needs to stay in longer we will explain why.

The nurses will monitor your drain output. We will try to remove your drains as soon as possible. Feel free to ask the team about this. If it needs to stay in longer we will explain why.

The nurses will give you all your medications. After you transfer to the ward, the nurses will explain your medications to you. They will help you to prepare for when you are discharged from hospital.

Progress on the critical care unit (ICCU)

**First 24hr in ICCU**	Oral diet and Tube feed ....<br>..<br>..<br>..<br>Number of walks____________ Distance____________<br>Time spent in chair__________(Hours/minutes)
**Day 1 in ICCU**	Oral diet and Tube feed ....<br>..<br>..<br>..<br>Number of walks____________ Distance____________<br>Time spent in chair__________(Hours/minutes)
**Day 2 in ICCU**	Oral diet and Tube feed ....<br>..<br>..<br>..<br>Number of walks____________ Distance____________<br>Time spent in chair__________(Hours/minutes)
**Day 3 in ICCU**	Oral diet and Tube feed ....<br>..<br>..<br>..<br>Number of walks____________ Distance____________<br>Time spent in chair__________(Hours/minutes)

Transferring to the transplant ward

When you no longer need the support of the critical care unit, you will transfer to the transplant ward, ward 38. This is an opportunity for you to really focus on your recovery and rehabilitation. You can play an important part in your own recovery by paying attention to your nutrition as well as your mobility.

The team will support you to try to achieve your daily targets. Don't worry if this isn't always possible, everyone is different and some days will be easier than others. The team will encourage you to be as active and engaged in your care as much as you are able. If something is worrying you, let the team know.

Ward 38 is a multi-organ transplant ward. The ward specialises in caring for patients with different types of organ transplants. As well as other liver transplant patients, you may see patients on the ward who have received kidney, islet cell, pancreas, heart and lung transplants. Visiting on ward 38 is usually Monday to Friday afternoons between 2pm and 4pm and in the evenings between 6pm and 8pm. At weekends it is 2pm -8pm. If your visitors find it difficult to come during these times, talk to the nurse in charge who may be able to help you. There is a consultant ward round every day and other members of the team are never far away if you need them.

Checklist for transfer

Do you know what your mobility targets are for today and tomorrow?

Are you managing to cough, take deep breaths and move?

Do you know what the plan is for your pain control for the next 24 hours?

Is there a plan for your catheter to be removed?

Have you told your family or friends that you are moving to the ward today?

If the answer to any of these questions above is no, let the transplant team know.

If you have questions for the transplant team, you might find it useful to write them down in this space.

....

....

....

....

....

....

....

....

....

Day of transfer to the transplant ward

You will usually be encouraged to drink to keep well hydrated. If you are struggling, let the nurses know. Sometimes we need you to aim for a particular amount each day, we will explain this to you if so.

Nutrition is an important part of your recovery. Your body needs more food than usual to heal. Eating snacks between meals can help. You may need feeding by a tube if you are not able to eat enough. Try to keep track and record what you are eating on the opposite page.

On the transplant ward, try to improve your mobility daily. This will help you to recover more quickly and help to prevent complications. The physiotherapy team will guide you (see pages 21-26). Use your journal to record your progress on the opposite page.

Your PCA will usually have stopped by now. Your tablets will help to keep you comfortable and mobile. It is important to be able to take deep breaths and cough. If you are struggling with this, talk to the team. We have many methods of pain relief we can try.

Your urinary catheter may have been removed by now. If you still have a catheter, we will try to remove it as soon as possible. Feel free to ask the team about this and they will discuss this with you. Ask the nurses for a leg bag to make it easier to move around.

Your drain/s may have been removed by now. If you still have a drain in, we will try to remove this as soon as possible. Feel free to ask the team about this. The nurses can help you to monitor your drain output.

The nurses will give you all the medications you need. Tomorrow, they will provide you with a medication card and help you to begin learning your new medications. This is an important step to help you to prepare for when you are discharged from hospital.

Progress on day of transfer to the transplant ward

Breakfast ....
....
....
....

Lunch ....
....
....
....

Evening meal ....
....
....
....

Snacks/supplements/tube feed
....
....
....
....
....
....
....
....
....
....
....
....
....
....
....

How many walks did you manage today? ☐ **Walks** ☐ **Metres**

How much time did you spend sitting in your chair? ☐ **Minutes**

What was your pain control like today? **(Please tick)**

Very good ☐ Good ☐ OK ☐ Poor ☐

If you have questions for the transplant team, you might find it useful to write them down in this space.

....
....
....

Day 1 on the transplant ward

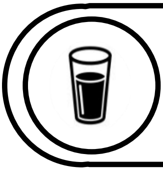

You will usually be encouraged to drink to keep well hydrated. If you are struggling, let the nurses know. Sometimes you need to aim for a particular amount each day. If so, we will explain why this is needed.

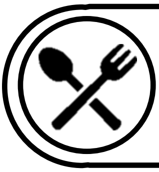

Your body needs more food than usual to heal. Snacking between meals can help. If you are not able to eat enough, you may still need feeding by a tube. Talk to the transplant team if you are struggling. Try to record what you are eating on the opposite page.

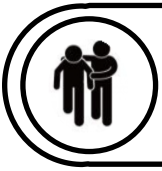

Try to increase your mobility today if you can. Some days may be easier than others. The nurses and physios will support you with your mobility programme (see pages 21-26). Use your journal to record your progress on the opposite page.

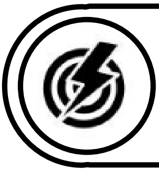

It is still important to be able to take deep breaths, cough and move around. If pain is making this difficult, talk to the team. We have many methods of pain relief we can try. Record your progress on the opposite page.

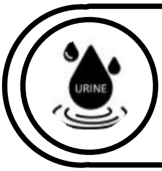

If you still have a drain in, we will try to remove this as soon as possible. Feel free to ask the team about this on the ward round. The nurses can help you to monitor your drain output. Ask the nurses for a safety pin so you can attach the bag to your clothes during the day.

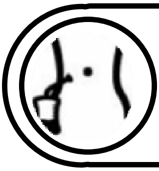

If you still need a catheter, the team will have discussed this with you. If you are not sure of the plan for this, just ask the transplant team on ward round. A leg bag can help to make it easier to move around – ask the nurses.

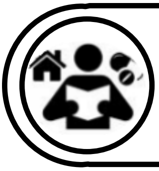

The nurses will give you a medication card and explain what each medication is for, what doses to take and when. Try to start learning about your tablets and getting them out for the nurses to check. This will help you to prepare for when you are discharged from hospital.

Progress on the transplant ward day 1

Breakfast ....
....
....
....

Lunch ....
....
....
....

Evening meal ....
....
....
....

Snacks/supplements/tube feed
....
....
....
....
....
....
....
....
....
....
....
....
....
....
....

How many walks did you manage today? ☐ **Walks** ☐ **Metres**

How much time did you spend sitting in your chair? ☐ **Minutes**

What was your pain control like today? (Please tick)

Very good ☐ **Good** ☐ **OK** ☐ **Poor** ☐

If you have questions for the transplant team, you might find it useful to write them down in this space.

....
....
....

Day 2 on the transplant ward

Keep drinking well to stay hydrated. If you are struggling, let the nurses know. Ice is usually available to keep your drinks cool. Cordial is also available, just ask the nurses.

Your body still needs more food than usual. Try to keep eating snacks between meals and keep track of what you are eating in the space opposite. Talk to the transplant team if you are finding this difficult. You may still need a feeding tube if you are not able to eat enough.

Try to follow the targets in your mobility plan (pages 21-26). This will help you to recover more quickly and help prevent complications. The physiotherapy and nursing team will support you. Use your journal to record your progress on the opposite page.

Ask for pain relief as and when you need it. It is important to manage your pain well so that you can move around and meet your mobility targets. Let the team know if you are struggling and record your progress on the page opposite.

If you still need a drain or urinary catheter, we will explain this to you. Feel free to ask the team about it. Ask the nurses to help you to monitor your output and help you to attach any drainage bags to your clothes to help you move around.

Use your medication to card to get your tablets out for the nurses to check. This will help you to prepare for when you are discharged from hospital. If you are finding this difficult, let the team know.

Today the nurses will provide you with information about how to look after your transplant and things to look out for to help prevent complications developing. If you have a support person, we recommend that they join you for this. Arrange a time with the nurses.

Progress on the transplant ward day 2

Breakfast ....
....
....
....

Lunch ....
....
....
....

Evening meal ....
....
....
....

Snacks/supplements/tube feed
....
....
....
....
....
....
....
....
....
....
....
....
....
....

How many walks did you manage today? ☐ **Walks** ☐ **Metres**

How much time did you spend sitting in your chair? ☐ **Minutes**

What was your pain control like today? (Please tick)

Very good ☐ **Good** ☐ **OK** ☐ **Poor** ☐

If you have questions for the transplant team, you might find it useful to write them down in this space.

....
....
....

Day 3 on the transplant ward

Keep drinking well to stay hydrated. If you are struggling, let the nurses know. Ice is usually available to keep your drinks cool. Cordial is also available, just ask the nurses.

Your body still needs more food than usual. Try to keep eating snacks between meals and keep track of what you are eating in the space opposite. Talk to the transplant team if you are finding this difficult. You may still need a feeding tube if you are not able to eat enough.

Keep following your mobility plan (pages 21-26). The physiotherapy team will give you safe exercises for you to try that you can carry on with at home. It is important to try to build up your strength and fitness levels safely to help keep you fit and well after the transplant.

Continue to ask for pain relief as and when you need it. It is important to be able to move around comfortably and meet your mobility targets. Let the team know if you feel your pain is not being well managed and record your pain feedback on the opposite page.

If you still need a drain or urinary catheter, we will explain this to you. Feel free to ask the team about it. Ask the nurses to help you to monitor your output and help you to attach any drainage bags to your clothes to help you move around.

Use your medication to card to get your tablets out ready for the nurses to check. This will help you to prepare for when you are discharged from hospital. If you are finding this difficult, let the team know.

An essential part of preparing for discharge from hospital is being informed about how to look after your transplant and help prevent complications. Please arrange a time to discuss this with the nurses if you have not already, or if you still have unanswered questions.

Progress on the transplant ward day 3

Breakfast ....
....
....
....

Lunch ....
....
....
....

Evening meal ....
....
....
....

Snacks/supplements/tube feed
....
....
....
....
....
....
....
....
....
....
....
....
....
....

How many walks did you manage today? ☐ **Walks** ☐ **Metres**

How much time did you spend sitting in your chair? ☐ **Minutes**

What was your pain control like today? (Please tick)

Very good ☐ **Good** ☐ **OK** ☐ **Poor** ☐

If you have questions for the transplant team, you might find it useful to write them down in this space.
....
....
....

Day 4 on the transplant ward

Keep drinking well to stay hydrated. If you are struggling, let the nurses know. Ice and cordial is usually available on the ward to help.

Your body still needs more food than usual. Try to keep eating snacks between meals and record what you are eating on the opposite page. Talk to the transplant team if you are finding this difficult. You may still need a feeding tube if you are not able to eat enough.

Keep following your mobility plan (pages 21-26). Don't forget that the physiotherapy team can give you exercises and advice about how to continue your recovery after discharge. It is important to try to be as active and healthy as you can to get the best out of your new liver.

Continue to ask for pain relief as and when you need it. It's important to be able to move around comfortably and meet your mobility targets. Let the team know if you feel your pain is not being well managed and record your pain feedback on the opposite page.

If you still need a drain or urinary catheter, ask the team about it. If you think you may need to leave hospital with a drain or catheter still in, ask the nurses to show you how to monitor your output and care for your tubes.

Keep using your medication to card to get your tablets out ready for the nurses to check. This will help you to prepare for when you are discharged from hospital. If you need your card rewriting or you are finding any of this difficult, let the team know and they can help.

Do you still have questions about how to care for your new liver and things to look out for to help prevent complications? If you do, try to arrange a time with the nurses. This is an essential part of preparing you for discharge from hospital.

Progress on the transplant ward day 4

Breakfast ....
....
....
....

Lunch ....
....
....
....

Evening meal ....
....
....
....

Snacks/supplements/tube feed
....
....
....
....
....
....
....
....
....
....
....
....
....
....
....

How many walks did you manage today? ☐ **Walks** ☐ **Metres**

How much time did you spend sitting in your chair? ☐ **Minutes**

What was your pain control like today? (Please tick)

Very good ☐ **Good** ☐ **OK** ☐ **Poor** ☐

If you have questions for the transplant team, you might find it useful to write them down in this space.
....
....
....

Day 5 on the transplant ward and beyond

Your nutrition is still important as you continue to recover. When you leave hospital, you need to continue to eat plenty of nourishing food. It is important to keep track of your weight and notice if you are losing or gaining weight. You are at higher risk of food poisoning, so you need to be careful with some foods. The nursing team talk to you about this when they go through the post-transplant booklet. This will help you learn to identify which foods to take extra care with or avoid. If you are unsure about this, talk to the team before you leave hospital.

It is important to think about how you will continue your recovery when you leave hospital. Do you have a plan for staying active and building up your strength? Talk to the physiotherapy team about this. It is important to get the best out of your new liver.

As you prepare to leave hospital, try to think about the pain medications you may need to take with you. Is your pain under control? Do you have a plan to reduce the amount of pain medications you are taking? Talk to the team if you are worried.

As you prepare to leave hospital, make sure you are confident in taking your medications at the correct time. Is your medication card up-to-date? Are you worried about remembering when to take your medications? Talk to us before you leave hospital so we can help.

Do you still have questions about how to care for your new liver and things to look out for to help prevent complications? If so, talk to the nurses and arrange a time to talk with them. This is an essential part of helping you prepare to leave hospital.

When you leave hospital, do you know when and where your next appointment is? Do you know what to do if you are worried about anything before then? Do you need transport? Do you have support at home? Discuss these things with the team as early as possible.

Progress on the transplant ward day 5

Breakfast ....
....
....
....

Lunch ....
....
....
....

Evening meal ....
....
....
....

Snacks/supplements/tube feed
....
....
....
....
....
....
....
....
....
....
....
....
....
....
....

How many walks did you manage today? ☐ **Walks** ☐ **Metres**

How much time did you spend sitting in your chair? ☐ **Minutes**

What was your pain control like today? (Please tick)

Very good ☐ Good ☐ OK ☐ Poor ☐

If you have questions for the transplant team, you might find it useful to write them down in this space.
....
....
....

Some patients may now be ready to be discharged from hospital, whilst others need to stay a little longer. Don't worry if this is the case, everyone is different. Please use the progress pages at the back of the journal to continue to record your progress (Page 31). It is still important to keep track of your nutrition and your mobility progress each day.

When you do leave hospital, you may also find it useful to use these blank pages to help you monitor your progress in the early days after discharge.

When you have left the hospital, don't forget, you are not alone. If you are worried about something before your next appointment, you can still contact the transplant team by calling the following numbers;

Mon-Fri 0830-1630 – Call the transplant coordinators on ……………… or …………………..

Out of hours and weekends – Call ward …. on …………………

Do you know when and where your follow up appointment is? (You can write it down here)

Date:_ _ _ _ _ _ _ _ _ _ _ _ _ _ _ Time:_ _ _ _ _ _ _ _

Place: Main outpatients
IOT outpatients
Other_ _ _ _ _ _ _ _ _ _ _ _ _ _

When you are ready to leave hospital after a liver transplant, it is common to feel a little worried about how you will manage without the constant access to health professionals. If there is anything in particular that is worrying you, talk to the team before you leave.

Leaving the hospital is the start of a long journey of recovery, often with some challenges along the way. Having a transplant can be an emotional experience and the transplant medications can also affect your mood. There are lots of support services available, offering a wide range of practical, social and emotional support including dedicated counsellors.

We have listed some links you may find useful below;

British Liver Trust
https://www.britishlivertrust.org.uk or call 0800 6527330

LiverNorth
https://www.livernorth.org.uk or call 0191 3702961

NHSBloodandTransplant
https://www.nhsbt.nhs.uk/organ-transplantation/liver

Mobility Programme

The first 24hr

- Breathing exercises
- Stand and march
- Try to sit out

Daily in ICCU

Breathing exercises

Sitting out

meals

Aim for ☐ hr per day

Walks

Aim for ☐ walks

Target distance ☐ metres

Daily in ICCU

Breathing exercises

Sitting out

meals

Aim for ☐ hr per day

Walks

Aim for ☐ walks

Target distance ☐ metres

Daily in ICCU

Breathing exercises

Sitting out

meals

Aim for ☐ hr per day

Walks

Aim for ☐ walks

Target distance ☐ metres

Day Of transfer

Breathing exercises

Try to sit out

meals

Aim for ☐ hr per day

Walks

Aim for ☐ walks

Target distance ☐ metres

Day 1 On the ward

Sitting out

meals

Aim for ☐ hr per day

Walks

Aim for ☐ walks

Target distance ☐ metres

Exercise Programme

Day 2 On the ward

Sitting out

meals

Aim for ☐ hr per day

Walks

Aim for ☐ walks

Target distance ☐ metres

Exercise Programme

Day 3 On the ward

Sitting out

meals

Aim for ☐ hr per day

Walks

Aim for ☐ walks

Target distance ☐ metres

Exercise Programme

Sitting out

meals

Aim for ☐ hr per day

Walks

Aim for ☐ walks

Target distance ☐ metres

Exercise Programme

Sitting out

meals

Aim for ☐ hr per day

Walks

Aim for ☐ walks

Target distance ☐ metres

Exercise Programme

Sitting out

meals

Aim for ☐ hr per day

Walks

Aim for ☐ walks

Target distance ☐ metres

Exercise Programme

Sitting out

meals

Aim for ☐ hr per day

Walks

Aim for ☐ walks

Target distance ☐ metres

Exercise Programme

Individual exercises

Exercises for ongoing rehabilitation

Drain

A tube coming from the transplant wound to remove blood and fluid that collects from the operation. It is connected to a bag that can be emptied so that the fluid volumes can be measured.

Education session

This is delivered by the nurses to give you information and help you better understand how to care for your new liver. This will include signs and symptoms of potential complications as well as things you should try to avoid. They can put you in touch with other professionals if you need help with finances, work or have other concerns.

Dietitian

Dietitians are qualified health professionals that assess, diagnose and treat dietary and nutritional problems. The dietitian you will see specialises in managing patients before during and after liver transplantation. They will help you to meet your nutritional needs as you recover from your transplant. This is an essential part of your transplant journey and can help you to recover more quickly and prevent complications.

ERAS (Enhanced recovery after surgery)

The name of the programme we are following to better support you as you recover from your operation.

Heat Pack
Heat packs are gel filled packs that can be heated (or cooled). By placing them on the affected area, they can help with post-operative pain. Avoid applying the pack directly to the skin or keeping them on too long, (the nurses can guide you with this).

ICCU Integrated critical care unit (ward 37)
Ward 37 or ICCU is the ward where patients are cared for immediately following a liver transplant. The nurses and doctors there specialise in looking after patients who are recovering from major surgery. On this ward they can monitor patients very closely and have access to lots of specialist tests and equipment to help support you if needed.

Medication card
You will be provided with a card that has a list of your medications. This includes the name of the medication, the dose you take, when to take it and what the medication is for. The nurses will guide you in how to use this card to identify your medications.

Nasogastric tube
A nasogastric (NG) tube is a plastic tubing device that sits in your nose and passes directly into the stomach. This can sometimes be used to help with the removal of stomach

contents after the operation or to give you fluids, medications and liquid food (*see tube feed*).

Nutrition

The process of providing or obtaining the food necessary for health and growth. This is particularly important when recovering from a liver transplant. Failing to obtain enough nutrients causes malnutrition. This can lead to complications such as problems with wound healing and can prolong your recovery time.

Physiotherapy mobility programme

A programme designed to help restore, maintain and make the most of a patient's mobility, function and well-being. After your transplant, the physiotherapists will assess you and provide you with a mobility programme. This is an important part of your recovery. They will take into account your level of fitness before transplant as well as your ability after the operation to make sure that the programme is right for you.

PCA = Patient controlled analgesia

This machine provides you with a painkiller called Fentanyl. This attaches to a cannula and enters the body through your veins. A dose is given each time you press the button. There is a safety mechanism, which means you will only receive a dose once every 5 mins regardless of how often you press it. This usually stops around 24 hours after surgery but can vary between patients.

Transplant team

A team of health professionals who specialise in caring for patients before, during and after liver transplantation. This includes Transplant surgeons, Liver medicine doctors, dietitians, physiotherapists, social workers, nurses and others.

Tube feed

When you are not able to get enough nutrition by eating and drinking, it may be necessary to support you with liquid nutrition given via a tube inserted into your nose (*see Nasogastric tube)*

Urinary Catheter

A urinary catheter is a thin tube that is inserted into the tube that carries urine out of the bladder. This usually happens during the liver transplant operation, when you are asleep and helps to empty the bladder whilst you wake up and start to recover from the operation.

Ward 38

Ward 38 is an adult multi-organ transplant unit that specialises in caring for patients before and after transplant surgery. Patients you will find on the ward include those recieving: Heart, Kidney, Liver, Lung, Pancreas and islet cell transplants. They also care for heart failure patients with Left ventricular assist devices.

Spare progress page

Progress post-transplant (__ days since transplant)

Breakfast ....
....
....
....

Lunch ....
....
....
....

Evening meal ....
....
....
....

Snacks / supplements / enteral
....
....
....
....
....
....
....
....
....
....
....
....
....
....
....

How many walks did you manage today? ☐ **Walks** ☐ **Metres**

How much time did you spend sitting in your chair? ☐ **Minutes**

What was your pain control like today? (Please tick)

Very good ☐ **Good** ☐ **OK** ☐ **Poor** ☐

If you have questions for the transplant team, you might find it useful to write them down in this space.
....
....
....

Progress post-transplant (__ days since transplant)

Breakfast ....
....
....
....

Lunch ....
....
....
....

Evening meal ....
....
....
....

Snacks / supplements / enteral
....
....
....
....
....
....
....
....
....
....
....
....
....
....
....

How many walks did you manage today? ☐ **Walks** ☐ **Metres**

How much time did you spend sitting in your chair? ☐ **Minutes**

What was your pain control like today? (Please tick)

Very good ☐ Good ☐ OK ☐ Poor ☐

If you have questions for the transplant team, you might find it useful to write them down in this space.
....
....
....

Progress post-transplant (__ days since transplant)

Breakfast ....
....
....
....

Lunch ....
....
....
....

Evening meal ....
....
....
....

Snacks / supplements / enteral
....
....
....
....
....
....
....
....
....
....
....
....
....
....
....

How many walks did you manage today? ☐ **Walks** ☐ **Metres**

How much time did you spend sitting in your chair? ☐ **Minutes**

What was your pain control like today? (Please tick)

Very good ☐ Good ☐ OK ☐ Poor ☐

If you have questions for the transplant team, you might find it useful to write them down in this space.
....
....
....

Progress post-transplant (__ days since transplant)

Breakfast ………………………………………

………………………………………………………

………………………………………………………

………………………………………………………

Lunch ………………………………………

………………………………………………………

………………………………………………………

………………………………………………………

Evening meal ………………………………

………………………………………………………

………………………………………………………

………………………………………………………

Snacks / supplements / enteral

………………………………………………………

………………………………………………………

………………………………………………………

………………………………………………………

………………………………………………………

………………………………………………………

………………………………………………………

………………………………………………………

………………………………………………………

………………………………………………………

………………………………………………………

………………………………………………………

………………………………………………………

………………………………………………………

………………………………………………………

How many walks did you manage today? ☐ **Walks** ☐ **Metres**

How much time did you spend sitting in your chair? ☐ **Minutes**

What was your pain control like today? (Please tick)

Very good ☐ **Good** ☐ **OK** ☐ **Poor** ☐

If you have questions for the transplant team, you might find it useful to write them down in this space.

…………………………………………………………………………………………

…………………………………………………………………………………………

…………………………………………………………………………………………

Progress post-transplant (__ days since transplant)

Breakfast ....
....
....
....

Lunch ....
....
....
....

Evening meal ....
....
....
....

Snacks / supplements / enteral
....
....
....
....
....
....
....
....
....
....
....
....
....
....
....

How many walks did you manage today? ☐ **Walks** ☐ **Metres**

How much time did you spend sitting in your chair? ☐ **Minutes**

What was your pain control like today? (Please tick)

Very good ☐ **Good** ☐ **OK** ☐ **Poor** ☐

If you have questions for the transplant team, you might find it useful to write them down in this space.
....
....
....

Progress post-transplant (__ days since transplant)

Breakfast

Lunch

Evening meal

Snacks / supplements / enteral

How many walks did you manage today? ☐ **Walks** ☐ **Metres**

How much time did you spend sitting in your chair? ☐ **Minutes**

What was your pain control like today? (Please tick)

Very good ☐ Good ☐ OK ☐ Poor ☐

If you have questions for the transplant team, you might find it useful to write them down in this space.

Progress post-transplant (__ days since transplant)

Breakfast ....
....
....
....

Lunch ....
....
....
....

Evening meal ....
....
....
....

Snacks / supplements / enteral
....
....
....
....
....
....
....
....
....
....
....
....
....
....
....

How many walks did you manage today? ☐ **Walks** ☐ **Metres**

How much time did you spend sitting in your chair? ☐ **Minutes**

What was your pain control like today? (Please tick)

Very good ☐ **Good** ☐ **OK** ☐ **Poor** ☐

If you have questions for the transplant team, you might find it useful to write them down in this space.
....
....
....

Progress post-transplant (__ days since transplant)

Breakfast ....
....
....
....

Lunch ....
....
....
....

Evening meal ....
....
....
....

Snacks / supplements / enteral
....
....
....
....
....
....
....
....
....
....
....
....
....
....
....

How many walks did you manage today? ☐ **Walks** ☐ **Metres**

How much time did you spend sitting in your chair? ☐ **Minutes**

What was your pain control like today? (Please tick)

Very good ☐ Good ☐ OK ☐ Poor ☐

If you have questions for the transplant team, you might find it useful to write them down in this space.
....
....
....
