# Supplementary material for: A review of enhanced recovery after surgery in kidney and liver transplantation and outline of the Newcastle ERAS protocols
Source: Front Transplant. 2026 May 28;5:1704028. doi: 10.3389/frtra.2026.1704028 (PMC13253640; doi:10.3389/frtra.2026.1704028)
Supplement: Supplementary file 2 [file Datasheet2.pdf]

The Newcastle upon Tyne Hospitals
NHS Foundation Trust

Enhanced recovery after surgery for kidney transplant recipients

Patient journal

Healthcare at its best
with people at our heart

x241922_NFH3975_p2_rr.indd 1 02/08/2022 16:06

x241922_NFH3975_p2_rr.indd 1 02/08/2022 16:06

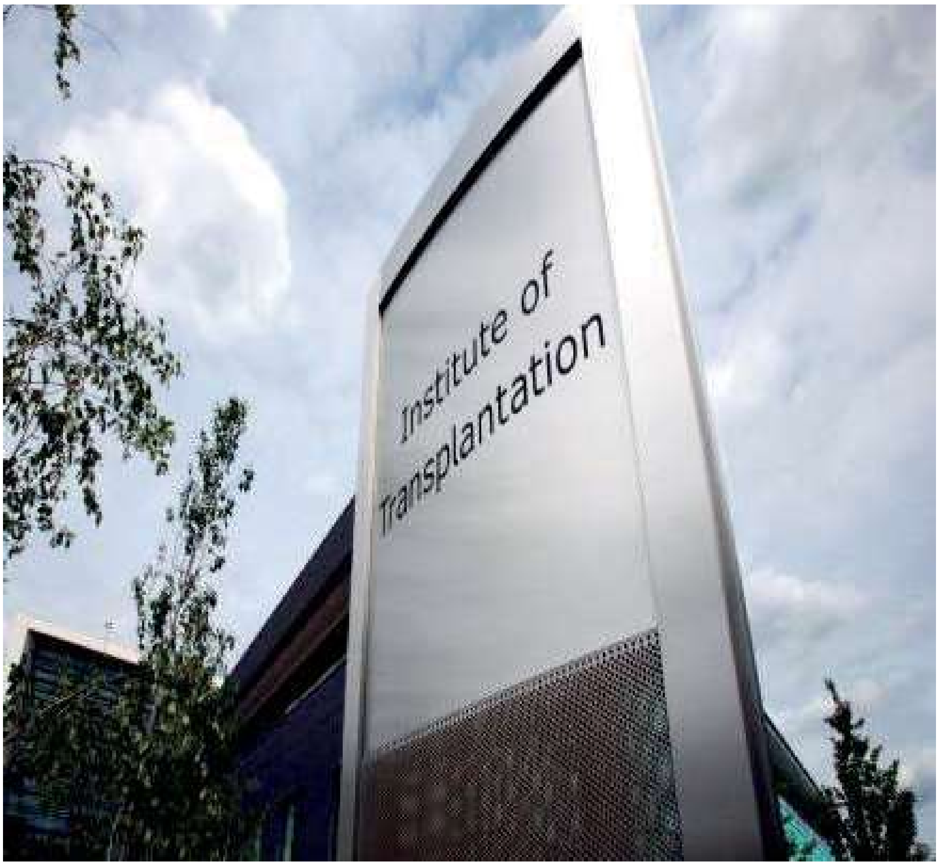

Please record your details below

Name: ....

Date of birth: ....

x241922_NFH3975_p2_rr.indd 2 02/08/2022 16:06

Introduction to our enhanced recovery programme

Enhanced recovery after surgery (ERAS) is designed to help you to recover more quickly from your transplant. Reaching certain targets can reduce the likelihood of complications and can help to improve the experience of your recovery.

To optimise your recovery after your transplant, it is important that you play an active role in your own care. We hope that this journal will help you to understand what to expect and help you to feel more involved in what happens during your transplant inpatient journey. The journal is yours to keep and will help you to record your progress. Relatives, friends and members of the transplant team can help you to complete it if you find this difficult. By completing this journal as you go, it will help us to support you and ensure that your recovery is as smooth as possible. There is a guide to fluid measurement on page 6 to help you. You can also find some useful explanations of terms on page 26.

Occasionally, some patients might struggle with elements of the programme. Don't worry if you don't meet all of your targets.

Every patient is different and the team will support you to achieve your targets at a pace that's right for you. The transplant team will adapt the programme where needed to ensure that you receive the high-quality care you need. We will not discharge you from hospital until both you and the transplant team feel that you are ready.

x241922_NFH3975_p2_rr.indd 3 02/08/2022 16:06

On admission for your transplant

- The team will ask you some questions to help plan for your needs when you go home.
- The physiotherapy team will see you either before or immediately after your transplant surgery and will explain your mobility targets to you. (See page 22)
- Along with your other routine tests, if you still pass urine, the team will perform a bladder scan on the ward. This will help us to decide how long your urinary catheter needs to stay in after the operation.
- If you have a relative or friend who can be a support person during your recovery, please arrange for them to be available (usually over the phone) in the afternoon on days 3 and 4 following your transplant. This is an important part of your recovery where the nurses will provide you with education on how to look after your transplant and the details of your medications.
- Please record the details of your support person below.

Named support person: ……………………………………………….……

Relationship: ……………………………………………………………….

Telephone number: ………………………………………………………

Date of transplant: ……………………………………………………...

x241922_NFH3975_p2_rr.indd 4 02/08/2022 16:06

During your hospital stay

Before surgery, you will need to fast. The team will guide you with timings. After the operation, you will be encouraged to eat and drink as soon as you feel able. We will try to stop your drip as soon as possible and will give you a fluid target to aim for.

The physiotherapy team will assess your mobility and provide you with a plan (A, B or C) to help you recover more quickly from the operation. Try to meet your daily targets and record your progress. We hope you will become more independent each day.

It is important to weigh yourself each morning to help us monitor your fluid balance. The team will help you with this as needed. We hope you will become more independent with this as you recover.

Your pain catheter will help to keep you comfortable for the first few days after surgery. We will try to stop your PCA after the first few hours to avoid unwanted side effects. The team will give you tablet pain relief as needed.

Where possible the surgeon will try to put just one drain in and we will aim to remove this as soon as it is safe to do so. The nurses will monitor the output and help you with this if the drain needs to stay in longer.

Your urinary catheter will be removed as soon as possible in the days after the operation, depending on certain criteria. We will help you to monitor and record your urine in your journal.

As soon as you feel able (usually on day two after the transplant), the team will provide you with a medication card and will support you to start taking your medications yourself with supervision.

The team will teach you and your support person about caring for your transplant in preparation for leaving hospital. If you need assistance with anything at home or transport to get home, let the team know as soon as possible.

x241922_NFH3975_p2_rr.indd 5 02/08/2022 16:06

Guide for measuring your drinks

Small beaker 170ml

Large beaker (blue) 240ml

Mug 190ml

Water Jug 1litre

x241922_NFH3975_p2_rr.indd 6 02/08/2022 16:06

After your surgery

We will stop the drip as soon as we can. You can start to eat and drink as soon as you feel able. If you are struggling, let the nurses know.

The team will help you sit out in your chair within the first 24 hours. The physiotherapy team will provide you with a mobility programme. It's really important to start this as soon as you are able (see pages 22-25).

The team will help you to weigh yourself before breakfast.

Your PCA and pain catheter will help to keep you comfortable. We will stop the PCA after the first few hours to avoid unwanted side effects. Talk to the nurses if you have pain.

The nurses will monitor and record your drain output.

The nurses will monitor and record your urine output.

The nurses will give you all your medications.

Let the team know if anything has changed with your home circumstances. Do you have someone to take you home and bring you to clinic appointments?

Progress after surgery

From tomorrow morning

We will ask you to start recording your fluid measurements.
We will also ask you to record your weight each morning before breakfast.

During the day

Each day, we will give you a fluid target to aim for. Please record it in the space provided.

In the evening

Please record your daily fluid target it in the space provided on each page. We will ask you to record how you are getting on each day. If you have any difficulties, concerns or have questions for the transplant team, you might find it useful to write them in the space provided each day.

....
....
....
....
....
....
....
....
....
....

x241922_NFH3975_p2_rr.indd 8 02/08/2022 16:06

Day 1 after surgery

If you still have a drip, it may stop today. You will be asked to drink and will be given a target to aim for. If you are struggling, let the nurses know. Start to record your fluid target and how much you drink on the opposite page.

The physiotherapy team will help you with your mobility programme (pages 22-25). This will usually include a number of walks. Try to sit out for each meal. Use your wall chart to keep track and record your progress on the opposite page.

The nurses will help you to weigh yourself before breakfast. Please record your weight on the opposite page.

Your PCA will usually have been stopped after the first twelve hours. Your pain catheter and oral medications will help to keep you comfortable and mobile. Talk to the nurses if you have pain.

The nurses will monitor and record your drain output.

The nurses will help you to start recording your urine output on the opposite page.

The nurses will give you all the medications you need.

Let the nurses know if anything has changed with your home circumstances. Do you have someone to take you home and bring you to clinic appointments?

x241922_NFH3975_p2_rr.indd 9 02/08/2022 16:06

Progress day 1

In the morning

The nurses will help you to start recording your drinks and your urine output in the box below.

Please record your weight this morning................................ kg
Please record today's fluid target.. ml

During the day

Please record today's fluid measurements

Drinks (ml)	Urine output (ml)
Total	Total

In the evening

Please record your progress today

Did you reach your fluid target? Yes/No
Did you manage to weigh yourself? Yes/No
How many walks did you manage today? ……….
How many times did you manage to sit in your chair? ....
How much time did you spend in your chair today? ....hr

If you have difficulties, concerns or have any questions for the transplant team, you might find it useful to write them down in this space.
....
....
....

x241922_NFH3975_p2_rr.indd 10 02/08/2022 16:06

Day 2 after surgery

Try to drink to your fluid target today. Please record your fluid target and the amounts you drink on the opposite page. The team will help you with this. Just ask.

Aim to sit in your chair for at least every meal and follow your mobility plan (see page 22-25). The team will support you with this. Use your wall chart to keep track and record your progress on the opposite page.

Please weigh yourself before breakfast and record your weight on the opposite page. Ask for help if needed.

Once empty, your pain catheter will be removed. Oral painkillers and heat packs are available to help keep you comfortable. Talk to the nurses if you have pain.

The nurses will monitor and record your drain output. Your drain may be removed today. Feel free to ask the team about this.

Your urinary catheter may be removed today. You will be given a jug to measure your urine. Please record your urine output on the opposite page. If it needs to stay longer, the nurses will change the catheter bag to make it easier for you to move around

The nurses will explain your medication card to you today and help you to start taking your own medications.

Let the team know if anything has changed with your home circumstances. Do you have a support person available to listen to your transplant education with you tomorrow?

Progress day 2

In the morning

How much did you drink yesterday? ....ml
How much urine did you pass yesterday? .... ml
Please record your weight this morning .... kg
Please record today's fluid target ....ml

During the day

Please record today's fluid measurements

Drinks (ml)	Urine output (ml)
Total	Total

In the evening

Please record your progress today

Did you reach your fluid target? Yes/No
Did you manage to weigh yourself? Yes/No
How many walks did you manage today? ……….
How many times did you manage to sit in your chair? ....
How much time did you spend in your chair today? ....hr

If you have difficulties, concerns or have any questions for the transplant team, you might find it useful to write them down in this space.

....
....
....

x241922_NFH3975_p2_rr.indd 12 02/08/2022 16:06

Day 3 after surgery

Try to drink to your fluid target today. Please record your fluid target and the amounts you drink on the opposite page. The team will help you with this. Just ask.

Aim to sit in your chair for at least every meal and follow your mobility plan (see page 22-25). The team will support you with this. Use your wall chart to keep track and record your progress on the opposite page.

Please weigh yourself before breakfast and record your weight on the opposite page. Ask for help if needed.

Continue to take oral painkillers as you need them. The nurses will help you and will record what you are taking. Heat packs are available. Talk to the team if you have pain.

If you still have a drain, it may be removed today. If you still need it, the nurses will show you how to look after it. Please start to record the output on the opposite page.

Please use your jug to measure your urine and record the output on the opposite page. If you still have a urinary catheter the nurses will help you to look after it and monitor your output.

Please continue to use your medication card to identify your medicines. The nurses will check they are correct before you take them.

Do you have a support person available for a video/phone call today? Ask the team to confirm timings for this. They will provide you with important education about caring for your transplant and how to manage when you leave hospital.

x241922_NFH3975_p2_rr.indd 13 02/08/2022 16:06

Progress day 3

In the morning

How much did you drink yesterday? ....ml

How much urine did you pass yesterday? .... ml

Please record your weight this morning .... kg

Please record today's fluid target ....ml

During the day

Please record today's fluid measurements

Drinks (ml)	Urine output (ml)
Total	Total

In the evening

Please record your progress today

Did you reach your fluid target? Yes/No
Did you manage to weigh yourself? Yes/No
How many walks did you manage today? ……….
How many times did you manage to sit in your chair? ....
How much time did you spend in your chair today? ....hr

If you have difficulties, concerns or have any questions for the transplant team, you might find it useful to write them down in this space.

....
....
....

x241922_NFH3975_p2_rr.indd 14 02/08/2022 16:06

Day 4 after surgery

Try to drink to your fluid target today. Please record your fluid target and the amounts you drink on the opposite page. The team will help you with this. Just ask.

Aim to sit in your chair for at least every meal and follow your mobility plan (pages 22-25). Use your wall chart and record your progress on the opposite page. The physio team will give you some exercises to help you continue your recovery at home.

Please weigh yourself before breakfast and record your weight on the opposite page. Ask for help if needed.

Continue to take oral painkillers as you need them. The nurses will help you and will record what you are taking. Heat packs are available. Talk to the team if you have pain.

If you still have a drain, it may be removed today. If you still need it, the nurses will show you how to look after it. Please continue to record the output on the opposite page.

Please use your jug to measure your urine and record the output on the opposite page. If you still have a urinary catheter the nurses will help you to look after it and monitor your output.

Please continue to use your medication card to identify your medicines. The nurses will check they are correct before you take them. We will start arranging a supply of all your medications ready for leaving hospital.

The team will continue with the education about caring for your transplant. We are preparing for when you leave hospital. It is very important to tell us about any worries or any help you need at home as soon as possible.

x241922_NFH3975_p2_rr.indd 15 02/08/2022 16:06

Day 4 after surgery

In the morning

How much did you drink yesterday? ....ml

How much urine did you pass yesterday? .... ml

Please record your weight this morning .... kg

Please record today's fluid target ....ml

During the day

Please record today's fluid measurements

Drinks (ml)	Urine output (ml)	Drain output (ml)
Total	Total	Total

In the evening

Please record your progress today

Did you reach your fluid target?	Yes/No
Did you manage to weigh yourself?	Yes/No
How many walks did you manage today?	……….
How many times did you manage to sit in your chair?	....
How much time did you spend in your chair today?	............hr

If you have difficulties, concerns or have any questions for the transplant team, you might find it useful to write them down in this space.

....

....

....

x241922_NFH3975_p2_rr.indd 16 02/08/2022 16:06

Day 5 after surgery (target day of discharge)

Try to drink to your fluid target and record this along with the amounts you drink on the opposite page. If you leave hospital today, please continue to record your drinks.

Continue to follow your mobility plan (pages 22-25). Use your wall chart and record your progress on the opposite page. The physio team will help you to plan your ongoing recovery at home.

Please weigh yourself before breakfast and record your weight on the opposite page. When you leave hospital, please continue to record your weight each day before breakfast.

Continue to take oral painkillers as you need them. The nurses will help you and will record what you are taking. Heat packs are available. Talk to the team if you have pain.

If you still have a drain, it may be removed today. If you still need it, the nurses will show you how to look after it. Please continue to record the output on the opposite page.

Please use your jug to measure your urine and record the output on the opposite page. The team will provide you with a jug to take home with you. If you still need a urinary catheter, the nurses will show you how to look after it and monitor your output yourself.

Please continue to use your medication card to identify your medicines. Make sure this is up to date before you leave hospital. A supply of all of your medications will be given to you to take with you when you leave hospital.

The team are preparing for you to leave hospital today. If you are ready. If you or your family are worried about anything, let us know.

x241922_NFH3975_p2_rr.indd 17 02/08/2022 16:06

Progress day 5

In the morning

How much did you drink yesterday? ....ml

How much urine did you pass yesterday? .... ml

Please record your weight this morning .... kg

Please record today's fluid target... ml

During the day

Please record today's fluid measurements

Drinks (ml)	Urine output (ml)	Drain output (ml)
**Total**	**Total**	**Total**

In the evening

Before you go home

- ☐ Do you have everything you need at home?
- ☐ Has transport been arranged to take you home?
- ☐ Have you arranged your transport to and from clinic?
- ☐ Is your medicine card up to date?
- ☐ If you have a dialysis line or PD catheter, has a removal date been arranged for you?

Your next follow up appointment is on ……/……/…… at …… : ……
We will call you on ……/……/…..

If you are worried about anything when you get home, please call us on 0191 2137538

x241922_NFH3975_p2_rr.indd 18 02/08/2022 16:06

Progress day 6

Please record yesterday's fluid measurements

- How much did you drink? ……………ml
- How much urine did you pass? ……………ml
- What was the drain output? ……………ml

Please record your weight this morning ……………..kg

Today's fluid target is... litres

....

....

Progress day 7

Please record yesterday's fluid measurements

- How much did you drink? .... ml
- How much urine did you pass?.......................... ml
- What was the drain output?............................... ml

Please record your weight this morning ……………..kg

Today's fluid target is...litres

....

....

Progress day 8

Please record yesterday's fluid measurements

- How much did you drink? ……………ml
- How much urine did you pass? ……………ml
- What was the drain output? ……………ml

Please record your weight this morning ……………..kg

Today's fluid target is..litres

....

....

Progress day 9

Please record yesterday's fluid measurements

- How much did you drink? .... ml
- How much urine did you pass?............................ ml
- What was the drain output?................................. ml

Please record your weight this morning ……………..kg

Today's fluid target is..litres

....

....

x241922_NFH3975_p2_rr.indd 20 02/08/2022 16:06

Progress day 10

Please record yesterday's fluid measurements

- How much did you drink? ....ml
- How much urine did you pass? ....ml
- What was the drain output? ....ml

Please record your weight this morning ....kg

Today's fluid target is.. litres

....

....

....

....

....

....

....

....

....

....

....

....

....

....

Healthcare at its best
with people at our heart

x241922_NFH3975_p2_rr.indd 21 02/08/2022 16:06

Mobility Programme

- Depending on the timing of your transplant, the physiotherapy team will see you just before your surgery or soon after, to assess your mobility and provide you with a recovery programme that's right for you.
- Patients are allocated into a mobility group (A, B, C) and this will determine your rehabilitation programme.
- The physiotherapy team will explain your targets and support you to achieve them. The ward team are also around to help you with this. It is important you try to play an active role in this to help you recover.
- We will provide you with a wall chart to keep track of your mobility and we will encourage you to record your daily progress in your journal.
- The physiotherapy team will provide you with some exercises so that you can continue to enhance your recovery after you leave hospital.

Your mobility group is

A B C

x241922_NFH3975_p2_rr.indd 22 02/08/2022 16:06

Mobility programme A

Day 1	Breathing exercises	2 to 4 Walks, 40m distance	Sitting out (x3)
Day 2	Breathing exercises	4 Walks, 90m distance	3-4 hr Sitting out (x3)
Day 3	4 to 6 Walks, 180m distance	3-4 hr Sitting out (x3)	Exercise Programme
Day 4	6 Walks, 180m distance	6-8 hr Sitting out (x3)	Exercise Programme
Day 5	6 Walks, 180m distance	6-8 hr Sitting out (x3)	Home Exercise Programme

x241922_NFH3975_p2_rr.indd 23 02/08/2022 16:06

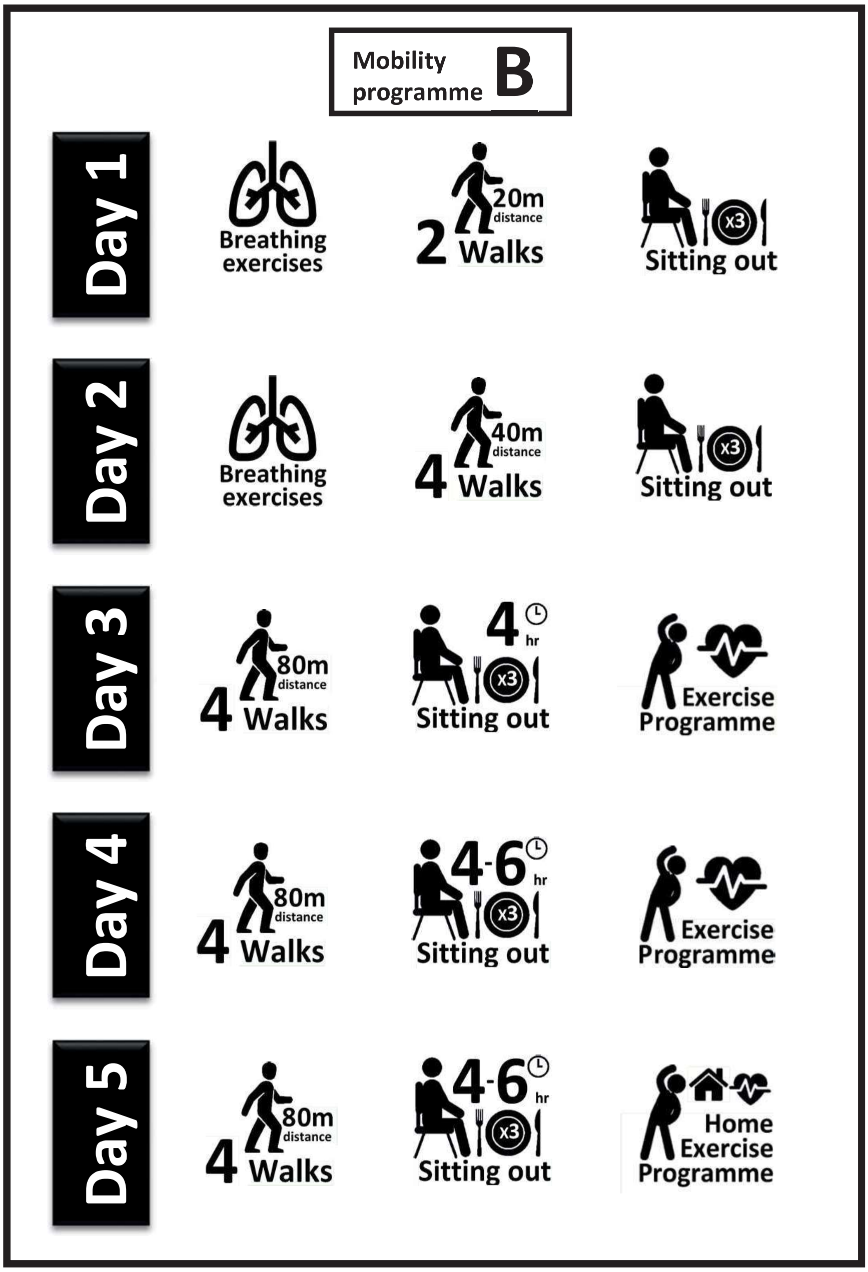

x241922_NFH3975_p2_rr.indd 24 02/08/2022 16:06

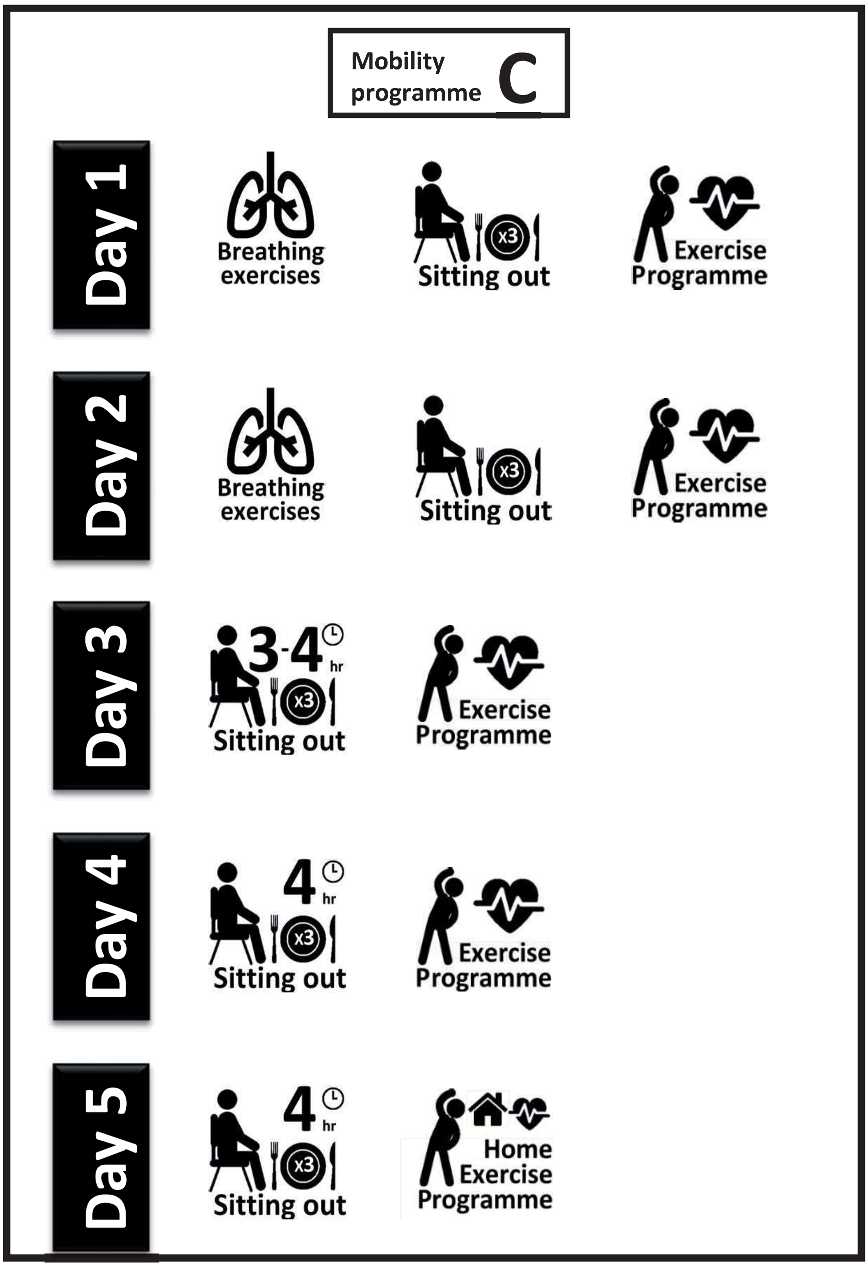

Mobility programme C
Day 1
Breathing exercises
x3
Sitting out
Exercise Programme
Day 2
Breathing exercises
x3
Sitting out
Exercise Programme
Day 3
3-4 hr
x3
Sitting out
Exercise Programme
Day 4
4 hr
x3
Sitting out
Exercise Programme
Day 5
4 hr
x3
Sitting out
Home Exercise Programme

Explanation of terms

- **Drain**

A tube coming from the surgical wound to remove blood and fluid that collects from the operation. It is connected to a bag that can be emptied so that the fluid volumes can be measured.

- **Education session**

This is delivered by the nurses to give you information and help you better understand how to care for your new kidney. This will include signs and symptoms of potential complications as well as things you should try to avoid. They can put you in touch with other professionals if you need help with finances, work or have other concerns.

- **ERAS = Enhanced recovery after surgery**

The name of the programme we are following to better support you as you recover from your operation.

- **Fluid target**

On the morning ward round each day, we will suggest a certain amount of fluid that we would like you to aim to drink over the course of the day. It is important to try to work towards this but don't worry if you don't reach the target. Talk to the transplant team if you are finding it difficult and they will help.

- **Medication card**

You will be provided with a card that has a list of your medications. This includes the name of the medication, the dose you take, when to take it and what the medication is for. The nurses will guide you in how to use this card to identify your medications.

x241922_NFH3975_p2_rr.indd 26 02/08/2022 16:06

Explanation of terms

- **Pain Catheter**

This is a tube inserted during the operation and sits around the muscle layer surrounding your new kidney. It provides a continuous supply of local anaesthetic to numb the area. The anaesthetic is stored in a ball that is attached to you. It usually lasts between 48 and 72 hours, then it can be removed. You may also hear people referring to this as a 'pain buster' or 'OnQ catheter'.

- **PCA = Patient controlled analgesia**

This is a machine that provides you with a painkiller called fentanyl. This attaches to a cannula and enters the body through your veins. A dose is given each time you press the button. There is a safety mechanism which means you will receive a dose once every five minutes regardless of how often you press it. This usually stops within the first twelve hours after surgery to avoid unwanted side effects.

- **Physiotherapy mobility programme**

A programme designed to help restore, maintain and make the most of a patient's mobility, function and well-being. After your transplant, the physiotherapists will assess you and provide you with a mobility programme with daily targets. This is an important part of your recovery. They will take into account your level of fitness before transplant as well as your ability after the operation to make sure that the programme is right for you.

x241922_NFH3975_p2_rr.indd 27 02/08/2022 16:06

x241922_NFH3975_p2_rr.indd 28 02/08/2022 16:06

x241922_NFH3975_p2_rr.indd 30 02/08/2022 16:06
