# Supplementary material for: A review of enhanced recovery after surgery in kidney and liver transplantation and outline of the Newcastle ERAS protocols
Source: Front Transplant. 2026 May 28;5:1704028. doi: 10.3389/frtra.2026.1704028 (PMC13253640; doi:10.3389/frtra.2026.1704028)
Supplement: Supplementary file 3 [file Datasheet3.pdf]

Newcastle upon Tyne NHS Foundation Trust ERAS in transplantation – supplementary material

1. Kidney transplant pathway supporting documents

1.1 Interactive patient journal

Refer to separate file for kidney transplant ERAS journal

1.2 Inpatient mobility infographic for kidney transplant recipients

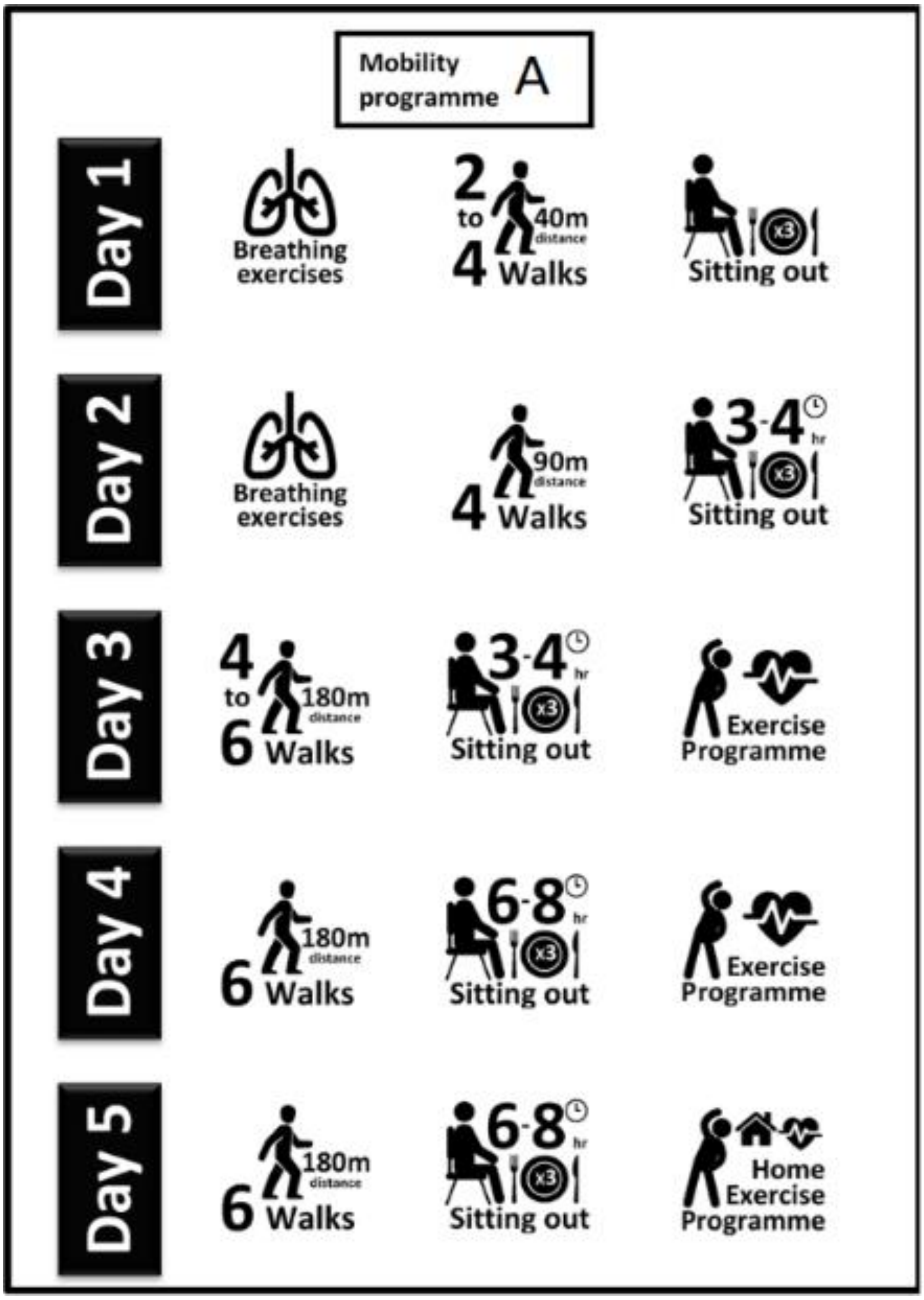

1.3 Inpatient mobility (kidney transplant)– patient record

Bed<br>___	**Enhanced recovery after surgery for renal transplant patients**<br>**Daily mobility progress chart**		
	Name:	Date of transplant: __/ __/2021	Mobility programme:

Post op Day	Sitting out in chair		Walks achieved					Exercise Programme
	For meals	Total time in chair	1	2	3	4	extra	
0	☐							
1	☐ ☐ ☐		☐	☐				
2	☐ ☐ ☐		☐	☐	☐	☐	☐	
3	☐ ☐ ☐		☐	☐	☐	☐	☐	
4	☐ ☐ ☐		☐	☐	☐	☐	☐	
5	☐ ☐ ☐		☐	☐	☐	☐	☐	

1.4 Flowchart for urinary catheter removal in kidney transplant recipients

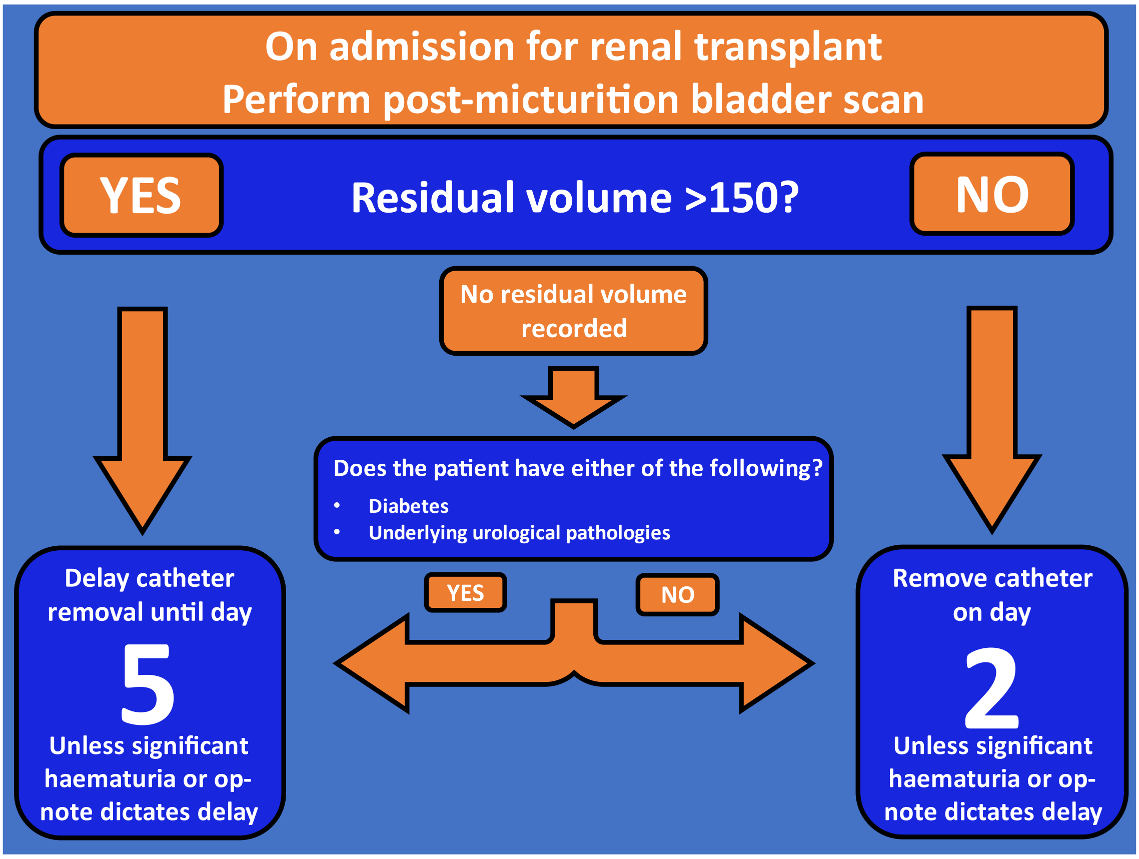

2. Liver transplant pathway supporting documents

2.1 Interactive patient journal

Refer to separate file for liver transplant recipient ERAS journal

2.2 Inpatient mobility infographic for liver transplant recipients

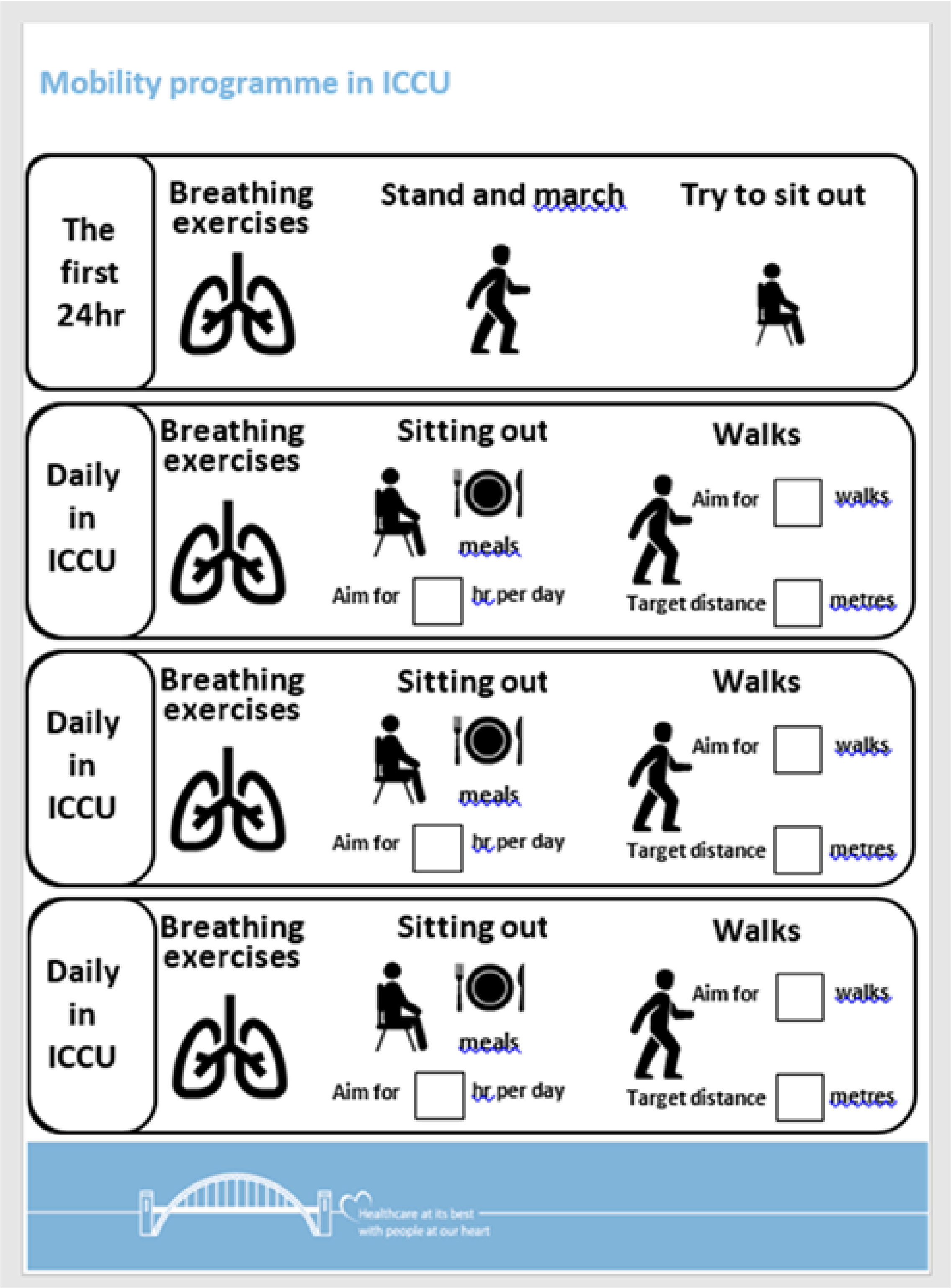

Mobility programme on the transplant ward

Day Of transfer

Breathing exercises

Try to sit out

meals

Aim for ☐ hr per day

Walks

Aim for ☐ walks

Target distance ☐ metres

Day 1 On the ward

Sitting out

meals

Aim for ☐ hr per day

Walks

Aim for ☐ walks

Target distance ☐ metres

Exercise Programme

Day 2 On the ward

Sitting out

meals

Aim for ☐ hr per day

Walks

Aim for ☐ walks

Target distance ☐ metres

Exercise Programme

Day 3 On the [illegible]

[No Title]

Sitting out

meals

Aim for ☐ hr per day

Walks

Aim for ☐ walks

Target distance ☐ metres

Exercise Programme
